# Peripheral metabolomic profiling reveals lipid and amino acid alterations associated with immuno-inflammatory responses in treatment-naïve late-onset Alzheimer’s disease

**DOI:** 10.3389/fnagi.2026.1858299

**Published:** 2026-06-23

**Authors:** Pei-Juan Wang, Xiao-Yu Yang, Lei Ji, Jia-Jia Shi, Xiang-Ming Cai, Peng Sun, He-Xu Liu, Fei Jiang, Fan Yang

**Affiliations:** 1 Department of Psychiatry, Nantong Fourth People’s Hospital, Nantong, China; 2 Lishui Key Laboratory of Brain Health and Severe Brain Disorders, Lishui Second People’s Hospital, Wenzhou Medical University, Lishui, China; 3 Department of Neurology, Affiliated Hospital of Zunyi Medical University, Zunyi, China; 4 Key Laboratory for the Genetics of Developmental and Neuropsychiatric Disorders (Ministry of Education), Bio-X Institutes, Shanghai Jiao Tong University, Shanghai, China

**Keywords:** Alzheimer’s disease, amino acids, Aβ, inflammation, late-onset, lipids, metabolomics, Tau

## Abstract

**Background:**

Immuno-metabolic dysregulation contributes to Alzheimer’s disease (AD) pathogenesis, yet the peripheral metabolic landscape and its interplay with neuroinflammation remain poorly characterized in treatment-naïve, late-stage patients. This study aimed to delineate plasma metabolic alterations and immuno-metabolic interactions in Chinese first-time outpatients with late-onset AD (CFTO-LOAD).

**Methods:**

Untargeted metabolomics and ELISA were applied to plasma from 35 CFTO-LOAD patients and 35 sex-matched cognitively healthy controls (CHCs) to quantify metabolites, cytokines (TNF-α, IL-17, IL-9), and soluble Aβ/Tau markers.

**Results:**

A total of 875 differentially abundant metabolites (DAMs) were identified in CFTO-LOAD, comprising 227 upregulated and 648 downregulated species (*P* < 0.05), predominantly lipids, fatty acids (e.g., dodecanoic acid, arachidonic acid), and amino acids (e.g., L-arginine, L-leucine). KEGG analysis revealed enrichment in fatty acid and amino acid metabolism, GABAergic synapse, and intestinal immune network pathways. CFTO-LOAD patients exhibited elevated pro-inflammatory cytokines TNF-α and IL-17 (*P*_*adj*_ < 0.05), reduced IL-9 (*P*_*adj*_ < 0.001), increased soluble p-Tau, p-Tau181, and p-Tau217 (*P*_*adj*_ < 0.01), and decreased Aβ42/Aβ40 ratio (*P*_*adj*_ < 0.001). Linear regression identified significant correlations between differential metabolites and immune/pathological markers, including positive associations of dodecanoic acid with TNF-α (*r* = 0.34, *P*_*adj*_ < 0.05) and arachidonic acid with Aβ42/Aβ40 ratio (*r* = 0.30, *P*_*adj*_ < 0.05), and negative associations of arachidonic acid with p-Tau217 (*r* = −0.43, *P*_*adj*_ < 0.01) and sphinganine 1-phosphate with TNF-α (*r* = −0.28, *P*_*adj*_ < 0.05).

**Conclusion:**

These findings characterize the peripheral immuno-metabolic landscape in treatment-naïve late-onset AD, identifying metabolic mediators that may mechanistically link neuroinflammation to Aβ and Tau pathology. This provides a foundation for biomarker development and therapeutic targeting in late-stage disease, pending independent validation.

## 1 Introduction

Alzheimer’s disease (AD) is a progressive neurodegenerative disorder characterized by declining memory, cognitive dysfunction, and impairments in language, comprehension, judgment, learning, and executive function (Ballard et al., 2011; Frisoni et al., 2025; Scheltens et al., 2016, 2021; Zheng and Wang, 2025). The global burden of AD is escalating rapidly: approximately 57.4 million individuals were affected by dementia in 2019, with projections reaching 152.8 million by 2050, disproportionately impacting low- and middle-income nations (GBD 2019 Dementia Forecasting Collaborators, 2022). The economic toll is staggering, with estimated treatment costs approaching 4.7 trillion US dollars by 2030 (Nandi et al., 2022). In China specifically, the 2024 national report documented 16.99 million existing AD and dementia cases—29.8% of the global total—amid rising incidence, prevalence, and mortality driven by accelerated population aging (Yang et al., 2024). This escalating crisis imposes severe burdens on families, healthcare systems, and geriatric rehabilitation infrastructure, underscoring the urgent need for novel biomarkers to enable early detection and effective intervention across disease stages.

Alzheimer’s disease pathogenesis involves interconnected molecular processes—Aβ deposition, Tau hyperphosphorylation, and neuroinflammation—that do not operate independently but rather form a self-amplifying pathogenic network (Heneka et al., 2015; Husain, 2022; Ossenkoppele et al., 2022). Genetic, epigenetic, and environmental risk factors converge upon this network (Kamatham et al., 2024; Liu et al., 2024; Scheltens et al., 2021; Zheng and Wang, 2025): advanced age remains the strongest risk factor for sporadic AD (Aries and Hensley-McBain, 2023), while the apolipoprotein E (APOE) ε4 allele confers the highest genetic susceptibility (Koutsodendris et al., 2022; Krishnamurthy et al., 2025; Serrano-Pozo et al., 2021). Critically, genome-wide association studies have identified numerous risk variants implicating not merely Aβ and Tau metabolism, but also lipid transport and innate immune pathways (Jansen et al., 2019; Kozlova et al., 2025; Kunkle et al., 2019; Novikova et al., 2021; Schwartzentruber et al., 2021), suggesting that metabolic-immune dysregulation represents a core pathogenic axis rather than a peripheral phenomenon.

Metabolomics—the comprehensive profiling of small-molecule metabolites—has revealed systematic metabolic reprogramming in AD patients and transgenic models (Batra et al., 2024; Dong et al., 2024; Horgusluoglu et al., 2022; Hüls et al., 2025; Meng et al., 2024; Milos et al., 2023; Montal et al., 2021; Pandey et al., 2024; Xu et al., 2012; Yi et al., 2017). Disturbances in energy metabolism, lipid homeostasis, and amino acid balance constitute early and persistent features of disease progression (Andersen et al., 2022; Lista et al., 2023; Tong et al., 2024; Ulland et al., 2017; Yin, 2023; Zhu et al., 2019). Notably, alanine-aspartate-glutamate metabolism is consistently disrupted across human and murine AD systems (Hernandez et al., 2025), implicating excitatory neurotransmitter balance and nitrogen disposal in pathogenesis. These metabolic alterations are not merely downstream consequences of neurodegeneration; emerging evidence positions them as active drivers of disease through their profound effects on immune cell function. The bidirectional coupling of immune and metabolic processes—termed immunometabolism—has emerged as a fundamental mechanism in AD (Asimakidou et al., 2025; Fairley et al., 2021; Gao et al., 2025; Hu et al., 2024; Peralta Ramos et al., 2025; Shippy and Ulland, 2020). Immune cells undergo dramatic metabolic reprogramming upon activation: to macrophages shift from oxidative phosphorylation aerobic glycolysis (the Warburg effect), while T cells modulate glutaminolysis and lipid synthesis to support proliferation and effector function (Kelly and O’Neill, 2015; Liu et al., 2021; MacIver et al., 2013; O’Neill et al., 2016; Shyer et al., 2020). Conversely, metabolites themselves serve as signaling molecules: short-chain fatty acids activate GPR43/FFAR2 on microglia, and branched-chain amino acids stimulate mTORC1 to drive inflammatory cytokine production (Cao et al., 2025; Dalile et al., 2019; Kim, 2021; Mann et al., 2024; Mukhopadhya and Louis, 2025). In AD, chronic neuroinflammation driven by activated microglia and astrocytes creates a pro-inflammatory metabolic milieu that impairs neuronal bioenergetics, while metabolic stress (e.g., mitochondrial dysfunction, insulin resistance) further primes immune cells toward inflammatory phenotypes, establishing a self-amplifying cycle that accelerates neurodegeneration and cognitive decline (Bach and Nguyen, 2026; Lonkar et al., 2025; Sadeghdoust et al., 2024).

Despite growing recognition of immunometabolic dysregulation in AD, critical gaps persist. Most metabolomics studies have examined patients receiving symptomatic treatments (cholinesterase inhibitors, memantine) or experimental therapies (anti-Aβ monoclonal antibodies, transcranial stimulation), which substantially alter peripheral metabolic profiles and immune parameters (Badhwar et al., 2020; Lista et al., 2023; Wang et al., 2026). Consequently, the “native” metabolic-immune state of untreated, late-stage AD—when pathological burden is maximal and compensatory mechanisms have failed—remains poorly characterized. In China, limited public awareness of AD and uneven healthcare distribution create a unique circumstance: a subset of patients progress to advanced disease without ever receiving standardized diagnosis or treatment (Zhi et al., 2025). These treatment-naïve individuals offer an unprecedented opportunity to study the unperturbed immunometabolic landscape of late-onset AD, identify biomarkers reflecting genuine disease biology rather than treatment effects, and elucidate regulatory mechanisms that could be targeted in advanced disease.

In this study, we applied untargeted metabolomics to plasma from CFTO-LOAD patients and CHCs to test the hypothesis that untreated late-stage AD exhibits a distinctive peripheral immunometabolic signature reflecting integrated lipid-amino acid-immune dysregulation. Our objectives: (1) to characterize peripheral metabolomic profiles in CFTO-LOAD; (2) to identify differentially altered lipids and amino acids associated with immuno-inflammatory responses; and (3) to explore potential metabolic-immune interactions that may contribute to LOAD pathogenesis. Our findings—extensive changes in fatty acids (e.g., arachidonic acid, dodecanoic acid) and amino acids (e.g., L-arginine, L-leucine), their enrichment in immunometabolic pathways, and their significant correlations with TNF-α, IL-17, p-Tau species, and Aβ42/40 ratio—support this integrated model. These results position peripheral immunometabolic dysregulation as a potential contributor to late-stage AD progression and identify candidate biomarkers and therapeutic targets warranting prospective validation.

## 2 Materials and methods

### 2.1 Study design and subject recruitment

This study was designed as a single-center, case-control observational study conducted at the Department of Psychiatry or Neurology, Nantong Fourth People’s Hospital, Jiangsu Province, China, between January 2022 and December 2024. The study protocol was reviewed and approved by the Ethics Committee of Nantong Fourth People’s Hospital (Approval No. 2022-K007), and all procedures were conducted in accordance with the ethical standards of the Declaration of Helsinki. Written informed consent was obtained from each participant or their legally authorized guardian prior to any study-related procedures.

#### 2.1.1 Inclusion criteria

Participants were enrolled into one of two groups based on their clinical and cognitive status. (1) CFTO-LOAD group: (i) Chinese Han ethnicity, aged ≥ 65 years; (ii) first-time outpatient visit to the neurology/psychiatry clinic with no prior exposure to anti-dementia medications, cholinesterase inhibitors, N-methyl-D-aspartate receptor antagonists, or any other psychotropic drugs; (iii) diagnosis of probable AD in accordance with the criteria established by the National Institute of Neurological and Communicative Disorders and Stroke-AD and Related Disorders Association (NINCDS-ADRDA) (McKhann et al., 1984) and the fourth edition of the Diagnostic and Statistical Manual of Mental Disorders (DSM-IV) (Dubois et al., 2021); (iv) age at symptom onset ≥65 years, consistent with the definition of late-onset AD; (v) abnormal findings on neuroimaging [computed tomography (CT) and/or magnetic resonance imaging (MRI)] indicative of medial temporal lobe and/or generalized cortical atrophy, without evidence of significant vascular lesions, space-occupying lesions, or normal-pressure hydrocephalus; (vi) global Clinical Dementia Rating (CDR) score ≥ 0.5; and (vii) availability of a reliable informant to provide collateral clinical history. (2) CHC group: (i) Chinese Han ethnicity, aged ≥ 65 years; (ii) matched to the CFTO-LOAD group with respect to sex; (iii) no subjective memory complaints and no objective cognitive impairment as assessed by the Chinese versions of the Mini-Mental State Examination (MMSE) and Alzheimer’s Disease-8 (AD8); (iv) normal performance on neuropsychological screening; (v) no significant abnormalities on structural neuroimaging (CT or MRI); and (vi) no first-degree relatives with a history of dementia.

#### 2.1.2 Exclusion criteria

Participants meeting any of the following criteria were excluded from enrollment: (i) early-onset AD (age at onset < 65 years) or familial AD with known autosomal dominant mutations in *APP*, *PSEN1*, or *PSEN2*; (ii) neurodegenerative or psychiatric disorders other than AD, including Parkinson’s disease, dementia with Lewy bodies, frontotemporal dementia, vascular dementia, major depressive disorder, bipolar disorder, or schizophrenia; (iii) diagnosed malignancies or active neoplastic disease; (iv) chronic metabolic or cardiovascular disorders, including hypertension (systolic blood pressure ≥ 140 mmHg and/or diastolic blood pressure ≥ 90 mmHg and/or use of antihypertensive medication), diabetes mellitus [fasting plasma glucose ≥ 7.0 mmol/L and/or glycated hemoglobin (HbA1c) ≥ 6.5% and/or use of hypoglycemic agents], dyslipidemia (total cholesterol ≥ 6.2 mmol/L, low-density lipoprotein cholesterol ≥ 4.1 mmol/L, triglycerides ≥ 2.3 mmol/L, and/or use of lipid-lowering drugs), or clinically significant hepatic or renal dysfunction; (v) autoimmune or chronic inflammatory diseases, including rheumatoid arthritis, systemic lupus erythematosus, inflammatory bowel disease, or multiple sclerosis; (vi) active infectious diseases, including bacterial, fungal, or viral infections, or a history of severe infection within the preceding 3 months; (vii) obesity, defined as a body mass index (BMI) ≥ 30.0 kg/m^2^; (viii) current smoking (≥1 cigarette per day for ≥1 year) or regular alcohol consumption (≥14 standard drinks per week for men or ≥7 standard drinks per week for women); (ix) treatment with immune-modulating drugs (including systemic corticosteroids, immunosuppressants, biologic agents, or non-steroidal anti-inflammatory drugs at anti-inflammatory doses) within 6 months prior to enrollment; and (x) participation in any other clinical trial within 30 days prior to enrollment.

#### 2.1.3 Sample size estimation

The sample size was determined a priori based on an estimated medium-to-large effect size for metabolomic differences between CFTO-LOAD patients and CHC individuals. Using G*Power software (v.3.1.9.7, Heinrich-Heine-Universität Düsseldorf, Germany), a power analysis for independent-samples *t*-tests with the following parameters was conducted: an anticipated Cohen’s d effect size of 0.60–0.75 (derived from published metabolomic studies comparing AD patients with controls), a two-tailed significance level of α = 0.05, and a target statistical power of 1−β = 0.80. The analysis indicated that a minimum of 29–36 participants per group would be required to detect significant between-group differences. To account for a potential attrition rate of approximately 10% and to ensure adequate statistical power for multivariate analyses and correlation assessments, we enrolled 35 participants in each group (total *n* = 70). This sample size satisfies the power requirement and is consistent with comparable metabolomic investigations in AD cohorts. Supplementary Table 1 provides detailed demographic and clinical characteristics for the enrolled CFTO-LOAD patients and CHC individuals.

### 2.2 Biospecimen collection

All participants were instructed to maintain an overnight fast of at least 8 h prior to blood collection, and all plasma samples were drawn in the morning (08:00–10:00) to minimize circadian variability. Water intake was permitted, but caloric and caffeine consumption was prohibited. From each participant, approximately 4.0 mL of peripheral blood was drawn into tubes with EDTA as an anticoagulant and subsequently centrifuged at 3,500 × *g* for 10 min (min) at 4 °C to isolate plasma. Following separation, the plasma was transferred into new 1.5 mL tubes and promptly cryopreserved using liquid nitrogen. Thereafter, all plasma samples were stored at −80 °C for subsequent analysis.

### 2.3 Untargeted metabolomics analysis

#### 2.3.1 Extraction of plasma total metabolites

After being thawed at 4 °C, 100 μL of each plasma sample was transferred to a new 2.0 mL centrifugation tube. Then, 100 μL of mixed internal standard solution and 400 μL of methanol (pre-chilled to −20 °C) were added to each sample, followed by vortexing for 60 s and centrifugation at 13,500 × *g* for 10 min at 4 °C. Next, 500 μL of the supernatant from each sample was transferred to another new 2.0 mL centrifugation tube. The samples were then vacuum-dried to complete dryness, reconstituted in 150 μL of 80% methanol, and re-centrifuged at 13,500 × *g* for 10 min at 4 °C. The supernatant was then transferred to a new centrifugation tube for LC-MS/MS analysis. Quality control (QC) samples were used to monitor analytical deviations (Sangster et al., 2006).

#### 2.3.2 Chromatographic separation assay

Chromatographic separation was conducted using a Thermo Ultimate 3000 system coupled with an ACQUITY UPLC^®^ HSS T3 column (2.1 × 150 mm, 1.8 μm particle size) (Waters Corporation Ltd., Milford, United States). The column temperature was maintained at 8 °C. Mobile phases were prepared by mixing 0.1% formic acid in ddH_2_O with acetonitrile, or alternatively, 5.0 mM ammonium formate in ddH_2_O with acetonitrile. Gradient elution was performed at a flow rate of 0.25 mL/min. After equilibration, a 2.0 μL aliquot of each sample was injected.

#### 2.3.3 Mass spectrometry assay

Mass spectrometry analysis was performed in accordance with established methodologies (Dunn et al., 2011). A Thermo Q Exactive mass spectrometer (ThermoFisher Scientific, United States) was employed to identify metabolites under both positive and negative electrospray ionization (ESI) modes. Simultaneous MS1 and MS/MS (Full MS-ddMS2 mode, data-dependent MS/MS) acquisition was used. The parameters were as follows: sheath gas pressure, 30 arbitrary (arb) units, auxiliary gas pressure, 10 arb units; spray voltage, 3.50 kV for ESI(+) and −2.50 kV for ESI(−); capillary temperature, 325 °C; MS1 scan range, mass/charge (m/z) 81–1,000; MS1 resolving power, 70,000 FWHM; number of data dependent scans per cycle, 3; MS/MS resolving power, 17,500 FWHM; normalized collision energy, 30 eV; dynamic exclusion time, automatic.

For data acquisition, metabolites were separated by column chromatography and subsequently detected by mass spectrometry. Each scan produced a mass spectrogram, with ion intensity on the y-axis and retention time on the x-axis. The ion with the highest intensity was continuously monitored to produce the base peak chromatogram (BPC).

#### 2.3.4 Data processing

Raw data were converted into mzXML format using the MSConvert tool from ProteoWizard (v3.0.8789) (Rasmussen et al., 2022). Following this, the XCMS package (v.3.12.0) (Navarro-Reig et al., 2015) in R was employed for peak detection, extraction, filtration, and alignment. After these steps, a data matrix was created, containing information like m/z ratios, retention times, and peak intensities.

#### 2.3.5 Data quality control

Initially, systematic errors were corrected using support vector regression with QC samples. Subsequently, data were filtered to remove low-quality information. Metabolites with a relative standard deviation (RSD) of over 30% in QC samples were removed, while those below this threshold were retained for further analysis.

### 2.4 Bioinformatic analysis

#### 2.4.1 Data normalization and gap-filling

Data normalization was conducted utilizing SIMCA-P software (v.14.1, Umetrics, Umea, Sweden) (Zhu et al., 2026). The parameters were configured as follows: the distance to the model was normalized using standard deviation units and weighted by modeling power; coefficients were scaled and centered; residuals/R2 were standardized; and the R2 type was set as R2-explained variation. To ensure robustness in the analysis, a Log_2_ transformation was applied to the raw metabolite data, using the formula Log_2_(raw value + 1) to stabilizes variance, reduces the impact of outliers, and retain information from low-abundance metabolite samples. This method helps stabilize variance, mitigate the influence of extreme values, and maintain information from low-abundance metabolite samples. Follow-up analyses were conducted using the transformed data. For missing dataset values, the k-nearest neighbors (k-NN) imputation method was employed (Lee and Styczynski, 2018). This method identifies the k-nearest neighbors for each metabolite and imputes missing data with their average value. Follow-up analyses were performed using the imputed data.

#### 2.4.2 Correction of confounders

The objective of this section is to systematically remove non-biological sources of variation—specifically demographic factors (sex and age) and technical artifacts (batch effects)—from the metabolomic dataset prior to downstream statistical analyses. Confounder correction is essential to ensure that observed metabolite differences between CFTO-LOAD patients and CHCs reflect disease-related biological perturbations rather than extraneous covariate-driven variation. The following three categories of confounders were addressed sequentially: sex, age, and batch effects.

Sex correction: Sex is a well-established source of inter-individual variation in human metabolomic profiles, with documented differences in lipid metabolism, amino acid homeostasis, and inflammatory mediator levels between males and females. To prevent sex-driven metabolite variation from confounding case-control comparisons, sex was treated as a covariate of no interest and statistically regressed out of the data. For each metabolite, a linear regression model was fitted with the raw metabolite abundance as the dependent variable and sex (coded as a binary variable: 0 = female, 1 = male) as the independent variable. The residuals that represent metabolite abundances with sex-associated variation removed, were retained as sex-corrected values and used as input for subsequent age correction.

Age correction: Advancing age is associated with substantial alterations in systemic metabolism, including changes in lipid composition, amino acid turnover, and oxidative stress-related metabolites. Because the CFTO-LOAD group was significantly older than the CHC group at baseline (mean age 80.40 ± 8.32 vs. 73.11 ± 4.01 years; *P* < 0.001; Supplementary Table 1), age was included as a covariate in the linear regression model to isolate disease-specific metabolite variations independent of age-related metabolic drift. Using the sex-corrected residuals as input, a linear regression model was fitted for each metabolite with age (in years, treated as a continuous variable) as the independent variable. The residuals that represent age- and sex-adjusted metabolite abundances and were carried forward as the corrected dataset for all subsequent statistical analyses.

Batch correction: Metabolomic data generated across multiple analytical batches are susceptible to systematic technical variation arising from instrument drift, reagent lot differences, operator variability, and run-order effects. Such batch effects, if unaddressed, can obscure true biological signals or generate false-positive associations. Prior to formal correction, principal component analysis (PCA) was performed on the sex- and age-adjusted data as an exploratory diagnostic step to visualize the distribution of samples across the first two principal components and to detect any clustering patterns indicative of batch-driven systematic variation. The ComBat method from the SVA package (v.3.42.0) (Leek et al., 2012) was applied to the sex- and age-adjusted metabolite matrix to remove batch effects. ComBat employs an empirical Bayes framework to estimate and adjust for location (mean) and scale (variance) shifts associated with batch membership while preserving biological variation related to the variable of interest (CFTO-LOAD vs. CHC status). The batch-corrected data matrix was then used as the final, fully adjusted dataset for all downstream differential abundance analyses, pathway enrichment analyses, and correlation analyses with immuno-inflammatory markers.

This sequential correction strategy was implemented to minimize the propagation of residual confounding at each step. The final corrected dataset contains metabolite abundances that are statistically independent of sex, age, and batch effects, thereby enhancing the internal validity and biological interpretability of our metabolomic findings.

#### 2.4.3 Multivariate statistical analysis

Before conducting multivariate analysis, metabolomics data were subjected to auto-scaling, which involves mean-centering and unit-variance scaling. This method ensures that each metabolite contributes equally to the analysis, independent of its initial concentration. Specifically, for each metabolite, the data were mean-centered by subtracting the mean value across all samples and then scaled to unit variance by dividing by the standard deviation across all samples. This scaling process adjusts metabolomics data to appropriate weights, enhancing the reliability and interpretability of results prior to multivariate analysis. In this study, multivariate statistical analysis was conducted using the Ropls package (v.1.22.0) (Thévenot et al., 2015) in R. Specifically, both unsupervised methods like PCA and supervised methods including partial least squares discriminant analysis (PLS-DA) and orthogonal PLS-DA (OPLS-DA) (Ma et al., 2021) were utilized to analyze all samples.

#### 2.4.4 Identification of differentially abundant metabolites

Differentially abundant metabolites (DAMs) were identified based on variable importance for the projection (VIP) scores exceeding 1.00 and *P*-values below 0.05. Metabolite identification was conducted by verifying molecular weights within a 15 ppm error range and subsequently matching against several databases, including HMDB^1^ (Wishart et al., 2007), mzCloud^2^ (Abdelrazig et al., 2020), MassBank^3^ (Horai et al., 2010), Lipid Maps^4^ (Sud et al., 2007), MoNA^5^, and Metlin^6^. Additionally, a custom database constructed by BioNovoGene Co., Ltd. (Suzhou, China) using MS/MS fragment data was employed. Identified metabolites were categorized using the Metabolon database. For differential analysis, Z-scores were computed based on metabolite levels relative to the control group’s mean and standard deviation, standardizing the data for comparison and quantifying relative metabolite levels. Pearson correlation coefficients were calculated using the base R “cor()” function (R v.4.0.3), with significance determined using the “cor.test()” function. MetPA within MetaboAnalyst 4.0^7^ was utilized to analyze metabolic pathways through hypergeometric tests. The ggplot2 package (v.3.3.4) in R was used to create box plots. Kyoto Encyclopedia of Genes and Genomes (KEGG) pathway enrichment analysis was performed using clusterProfiler (v.4.2.2) (Wu et al., 2021; Yu et al., 2012) in R.

#### 2.4.5 Agglomerate hierarchical clustering

Agglomerative hierarchical clustering was utilized to categorize and integrate samples. An agglomerative approach was employed to classify and merge samples. The Pheatmap package (v.1.0.12) (Gatto et al., 2015) in R was employed to determine relative metabolite levels. Distances were computed via a distance matrix, with samples clustered through average linkage clustering. Heatmaps were created using the Pheatmap package (v.1.0.12) (Gatto et al., 2015).

### 2.5 Measurement of plasma cytokine, chemokine, soluble Aβ, and Tau levels

The concentrations of selected cytokines, chemokines, and neurodegeneration-associated proteins were quantified in plasma samples using commercially available sandwich enzyme-linked immunosorbent assay (ELISA) kits, following the manufacturers’ standardized protocols with additional in-house quality control measures. The specific analytes measured included: (i) pro-inflammatory cytokines and chemokines: tumor necrosis factor-α (TNF-α), interleukin-17 (IL-17), IL-8, and IL-9; and (ii) Alzheimer’s disease-related biomarkers: soluble amyloid-β42 (Aβ42), amyloid-β40 (Aβ40), total phosphorylated Tau protein (p-Tau), Thr181-phosphorylated Tau (p-Tau181), and Thr217-phosphorylated Tau (p-Tau217) (Gonzalez-Ortiz et al., 2023; Karikari et al., 2020; Thijssen et al., 2021).

#### 2.5.1 ELISA kits and reagent specifications

All ELISA kits were purchased from HNYBio Co., Ltd. (Shanghai, China) and employed a sandwich immunoassay format with capture antibodies pre-coated onto 96-well polystyrene microplates and detection antibodies conjugated to horseradish peroxidase (HRP). The catalog numbers for the nine analytes were as follows: TNF-α (Cat. No. HB090-Hu), IL-17 (Cat. No. HB1984-Hu), IL-8 (Cat. No. HB1944-Hu), IL-9 (Cat. No. HB1943-Hu), Aβ42 (Cat. No. HB2062-Hu), Aβ40 (Cat. No. HB2063-Hu), p-Tau (Cat. No. HB1023-Hu), p-Tau181 (Cat. No. HB1024-Hu), and p-Tau217 (Cat. No. HB2598-Hu). The working concentration for all capture and detection antibody reagents was standardized at 20 ng/mL, as optimized and validated by the manufacturer.

#### 2.5.2 Assay procedure and quality controls

Plasma samples were thawed on ice, centrifuged at 1,000 × *g* for 10 min at 4 °C to remove particulate matter, and diluted 1:5 in assay diluent prior to analysis.

Blank controls: Two wells per plate were loaded with assay diluent only (without sample or standard) to assess background optical density (OD) signal. The mean blank OD was subtracted from all sample and standard OD values prior to concentration calculations.

Standard curves: Five-point serial dilutions of recombinant protein standards (supplied with each kit) were prepared in duplicate, spanning the dynamic range of each assay. A standard curve was accepted only if the coefficient of determination (R^2^) exceeded 0.99. The lower limit of detection (LLOD) and lower limit of quantification (LLOQ) were defined as the concentrations corresponding to mean blank OD plus 3 and 10 standard deviations, respectively.

Sample quantification: Concentrations were calculated by interpolating sample OD values against the assay-integrated standard curves using a four-parameter logistic (4-PL) regression model. Results were expressed in picograms per milliliter (pg/mL). Data acquisition and analysis were performed using Bio-Plex Manager software (version 6.0; Bio-Rad Laboratories, Hercules, CA, United States). Prior to statistical analysis, ELISA data were corrected for age using linear regression to exclude the confounding effect of the significant age difference between the CFTO-LOAD and CHC groups. Age-corrected residuals were used for all subsequent group comparisons and correlation analyses.

#### 2.5.3 Assay validation

The intra-assay precision (repeatability), assessed by measuring three quality control samples within the same plate, yielded coefficient of variation (CV) values ranging from 5% to 8%. The inter-assay precision (reproducibility), assessed by measuring the same quality control samples across three independent assay runs on different days, also yielded CV values ranging from 5% to 8%. These values are within the acceptable range for research-grade ELISA immunoassays and indicate robust assay performance.

### 2.6 Correlation analysis of DAMs with immuno-inflammatory markers and Alzheimer’s disease-related biomarkers

To explore potential mechanistic links between peripheral metabolic alterations and both systemic immuno-inflammatory status and core AD pathophysiology in our LOAD cohort. We selected DAMs—specifically amino acids and fatty acids identified in Section 2.3 and 2.4—for correlation analysis. Two categories of biomarkers were analyzed as correlates: (i) Immuno-inflammatory markers: TNF-α, IL-17, IL-8, and IL-9, and (ii) AD-related biomarkers: Soluble Aβ42, Aβ40, total p-Tau, p-Tau181, and p-Tau217. We explicitly distinguish between these direct and indirect relationship frameworks in interpreting correlation results: significant correlations with cytokines suggest metabolite-immune crosstalk potentially amenable to metabolic intervention; significant correlations with Aβ/Tau species suggest metabolite-neurodegeneration associations that may reflect either causal contributions or parallel consequences of disease progression. Prior to correlation analysis, the untargeted metabolomics data were corrected for age, sex, and batch effects as described in section “2.4.2 Correction of confounders,” and ELISA data were corrected for age as described in section “2.5 Measurement of plasma cytokine, chemokine, soluble Aβ, and Tau levels.” These confounder corrections ensure that the observed correlations reflect disease-specific metabolic–immune–pathological interactions rather than demographic artifacts.

#### 2.6.1 Correlation of DAMs with cytokines and chemokines

To assess relationships between DAMs and systemic inflammatory status, we performed pairwise correlation analyses between each differentially abundant amino acid and fatty acid (post-confounder correction, section “2.4.2 Correction of confounders”) and each cytokine/chemokine (TNF-α, IL-17, IL-8, IL-9). For metabolite-cytokine pairs with normal distributions (assessed via Shapiro-Wilk test), Pearson’s product-moment correlation coefficients (r) were computed. Statistical significance was set at *P* < 0.05, with Benjamini-Hochberg (BH) false discovery rate (FDR) correction applied across all metabolite-cytokine comparisons to control for multiple testing [FDR-adjusted P (*P*_adj_) < 0.05 considered significant].

#### 2.6.2 Correlation of DAMs with AD-related biomarkers (Aβ and tau species)

To assess relationships between peripheral metabolic alterations and CNS pathological burden, we performed pairwise correlation analyses between each DAM and each AD-related biomarker: Aβ42, Aβ40, the Aβ42/Aβ40 ratio, total p-Tau, p-Tau181, and p-Tau217. The Aβ42/Aβ40 ratio was included as an index of amyloid aggregation propensity and pathogenicity. The same statistical procedures described in section “2.6.1 Correlation of DAMs with cytokines and chemokines” were applied.

### 2.7 Statistical analysis

Metabolite intensity data were log_2_-transformed to stabilize variance and improve distributional normality. For high-dimensional metabolomics data, Welch’s *t*-tests were uniformly applied to all metabolite features for group comparisons. For low-dimensional clinical biomarkers (e.g., cytokine, chemokine, and AD-related marker levels after age correction), normality was assessed using the Shapiro-Wilk test; variables with normal distributions and homogeneous variances (assessed by Levene’s test) were compared using independent samples *t*-tests, those with normal distributions but unequal variances were analyzed using Welch’s *t*-tests, and non-normally distributed variables were compared using Mann-Whitney U-tests. For ELISA-derived biomarker data, *t*-tests were performed on age-corrected residuals to account for the significant baseline age difference between groups. Categorical variables (e.g., sex) were compared using Pearson’s chi-square tests or Fisher’s exact tests when expected cell frequencies were less than 5. The false discovery rate (FDR) for metabolomics data was controlled using the Benjamini-Hochberg (BH) method. Only an adjusted *P*-value (*P*_*adj*_) < 0.05 was considered statistically significant. Statistical analyses were conducted using SPSS V19.0 software (Chicago, IL, United States). Graphs were generated using GraphPad Prism V9.0 (GraphPad Software, San Diego, CA, United States).

## 3 Results

### 3.1 The peripheral systemic metabolic profile of CFTO-LOAD patients undergoes significant alterations

To characterize the native peripheral systemic metabolic profile of Chinese first-time outpatients with late-onset Alzheimer’s disease (CFTO-LOAD, abbreviated as AD), with particular emphasis on fatty acid and amino acid metabolism, we performed untargeted metabolomic profiling of plasma samples from 35 AD patients and 35 cognitively healthy controls (CHC) (detailed cohort information is provided in Supplementary Table 1).

**FIGURE 1:**
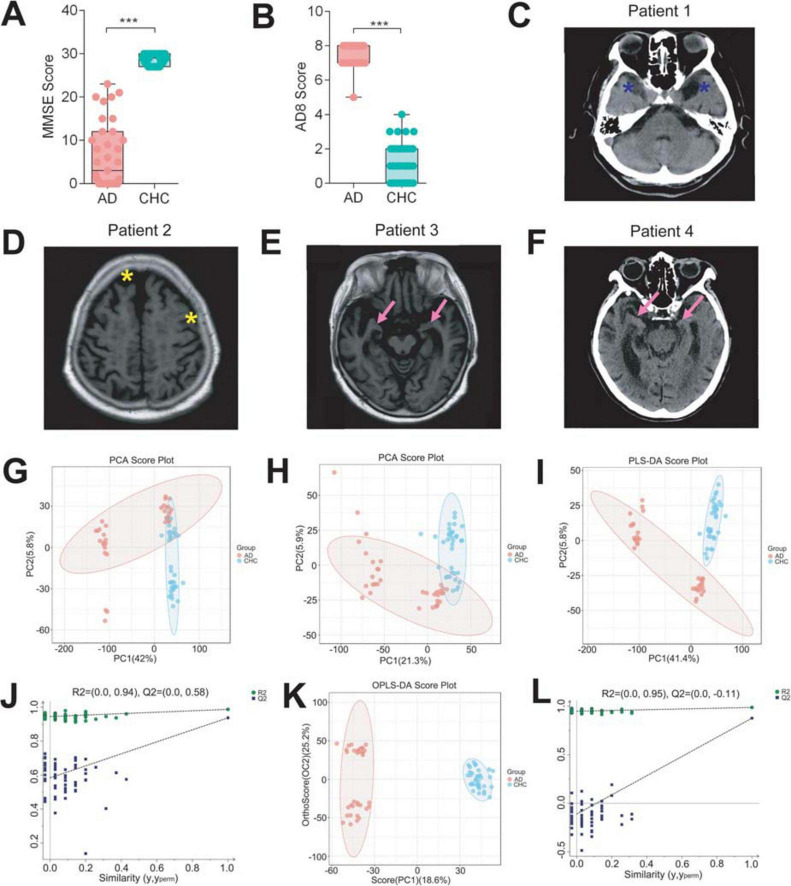
Clinical diagnosis of Alzheimer’s disease (AD) patients and multivariate statistical analysis of AD and cognitively healthy control (CHC) samples. **(A,B)** Box plots show MMESE and AD8 score of 35 AD patients and 35 CHCs, respectively. Samples between the two groups were compared using independent *t*-tests. *P*-values were false discovery rate (FDR)-corrected using BH method. ***P_*adj*_ < 0.001. **(C–F)** Representative brain CT and MRI images of AD patients. **(C)** Blue asterisks denote distinct atrophy of temporal lobe and expansion of temporal horn in the bilateral brain of AD patient 1. **(D)** Obvious atrophy of cerebral gyrus of AD patient 2, yellow asterisks indicate deepened sulcus. **(E,F)** Pink arrows highlight the significantly decreased transverse diameter of hippocampus in the bilateral brain of AD patient 3 and patient 4, respectively. **(G,H)** Principal Component Analysis (PCA) analysis results of 35 AD and 35 CHC samples in both cationic **(G)** and anionic **(H)** modes. **(I,J)** partial least squares discriminant analysis (PLS-DA) analysis results of 35 AD and 35 CHC samples in cationic mode. The model interpretability R2X = 0.526, and R2Y = 0.987, model predictability Q2 = 0.937. **(K,L)** Orthogonal partial least squares discriminant analysis (OPLS-DA) analysis results of 35 AD and 35 CHC samples in cationic mode. The model interpretability R2X = 0.526, and R2Y = 0.987, model predictability Q2 = 0.877. R2X/R2Y, cumulative model fit; Q2, predictive ability.

Prior to metabolomic analysis, we verified the clinical and neuroimaging distinctions between the two groups. The Mini-Mental State Examination (MMSE) scores demonstrated marked impairment in the AD group compared with the CHC group. The AD group exhibited a mean MMSE score of 6.43 ± 7.54, whereas the CHC group achieved a mean score of 28.46 ± 1.20. This difference was highly statistically significant (*P*_*adj*_ < 0.001, Figure 1A and Supplementary Table 1). The Alzheimer’s Disease-8 (AD8) scale, an informant-based measure of functional and cognitive decline, yielded complementary findings. The AD group demonstrated significantly elevated AD8 scores compared with the CHC group (AD: 7.66 ± 0.64; CHC: 1.29 ± 1.13; *P*_*adj*_ < 0.001, Figure 1B and Supplementary Table 1). Collectively, these cognitive assessments confirm that the AD group exhibited substantial cognitive impairment with corresponding functional disability, whereas the CHC group demonstrated intact cognitive performance across all domains. Structural neuroimaging via computed tomography (CT) and magnetic resonance imaging (MRI) revealed characteristic patterns of neurodegeneration in the AD group that were absent in the CHC group. Representative images are presented in Figures 1C–F with annotated markers indicating the affected anatomical regions. Blue asterisks denote significant bilateral temporal lobe atrophy with compensatory expansion of the temporal horns of the lateral ventricles, a hallmark feature of AD-associated medial temporal lobe degeneration (Figure 1C); Yellow asterisks indicate diffuse cerebral gyrus atrophy with concomitant deepening sulcus (Figure 1D). This pattern is consistent with the posterior cortical atrophy trajectory characteristic of typical amnestic AD; Pink arrows highlight the significant reduction in the transverse diameter of the bilateral hippocampus (Figures 1E, F), collectively confirming the clinical diagnosis of AD in these first-time outpatients.

Quality control assessment of the metabolomic data demonstrated robust analytical performance and clear metabolic divergence between the two groups. Unsupervised principal component analysis (PCA) revealed distinct separation of AD and CHC samples in both positive and negative ion modes (Figures 1G, H). Supervised partial least squares discriminant analysis (PLS-DA) and orthogonal PLS-DA (OPLS-DA) further confirmed significant group segregation in both ion modes (Figures 1I–L and Supplementary Figures 1A–D), reflecting pronounced inter-group metabolic differences with high intra-group homogeneity.

**FIGURE 2:**
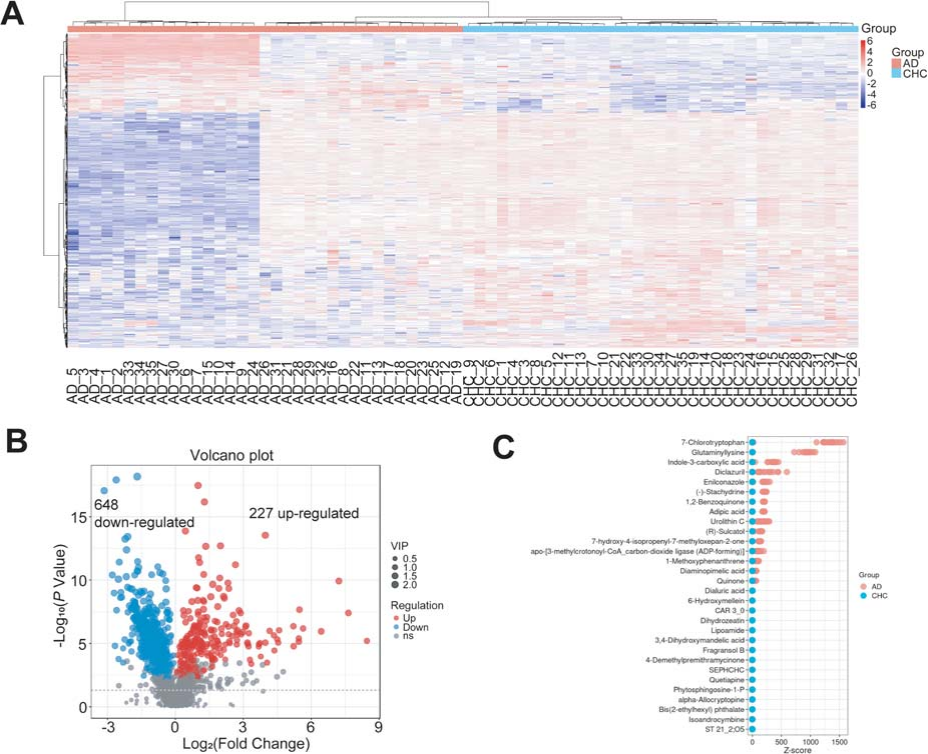
Identification of differentially abundant metabolites in plasma samples of Alzheimer’s disease (AD) patients compared to cognitively healthy control (CHC). **(A)** Heatmap indicates 875 differentially abundant metabolites (DAMs) were identified in plasma samples of AD patients compared to that of CHC. VIP > 1.00, and *P* < 0.05. **(B)** Volcano plot shows 227 significantly up-regulated and 648 down-regulated DAMs identified in AD samples. **(C)** Z-score plot exhibits the level changes of most significantly 15 up-regulated and 15 down-regulated DAMs.

To identify differentially abundant metabolites (DAMs) significantly associated with AD, we applied stringent filtering criteria (VIP > 1.0 and *P* < 0.05). Hierarchical clustering of the metabolic profiles revealed substantial alterations in the AD plasma metabolome compared with the CHC group (Figure 2A). In total, 875 DAMs were identified, comprising 227 significantly upregulated and 648 significantly downregulated metabolites (Figure 2B). Representative DAMs included significantly elevated levels of 7-Chlorotryptophan and Enilconazole in AD plasma (*P*_*adj*_ < 0.05). Conversely, Phytosphingosine-1-P, 3,4-Dihydroxymandelic acid, and Quetiapine were significantly decreased (*P*_*adj*_ < 0.05; Figure 2C). These findings indicate widespread disruption of tryptophan metabolism and sphingolipid signaling in the peripheral circulation of treatment-naïve AD patients.

### 3.2 Identification of differential amino acids and fatty acids related to CFTO-LOAD

**FIGURE 3:**
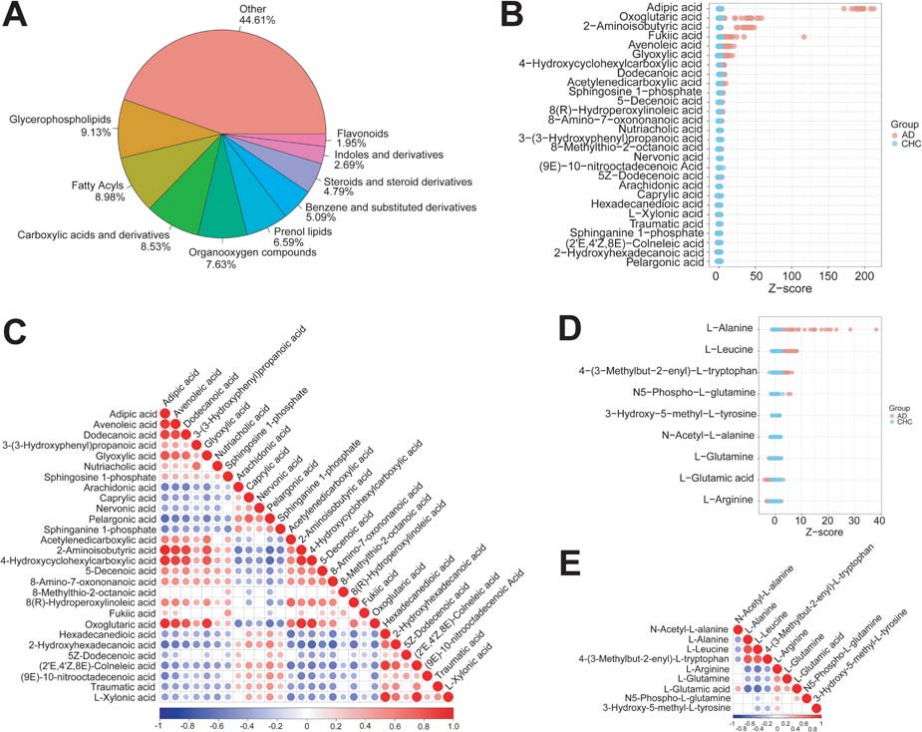
Metabolic profiles of fatty acid and amino acid were significantly changed in Alzheimer’s disease (AD) patients compared to cognitively healthy control (CHC) group. **(A)** Pie chart shows the classification of all differentially abundant lipids/fatty acids and their derivatives in AD patients. **(B)** Z-score plot demonstrates the concentration changes of 28 representative fatty acids and their derivatives in plasma of AD patients relative to CHC. **(C)** Pearson’s correlation analysis results show significant correlations between the level changes of 28 representative fatty acids and their derivatives. **(D)** Z-score plot shows the abundance changes of nine representative amino acids and their derivatives in plasma of AD patients compared to CHC. **(E)** Pearson’s correlation analysis results show significant correlations between the concentration changes of nine representative amino acids and their derivatives. **(C,E)** Positive and negative correlation was denoted by red and blue, respectively.

Chemical taxonomy of the 875 DAMs revealed that the largest proportions belonged to glycerophospholipids (9.13%), fatty acyls (8.98%), carboxylic acids and derivatives (8.53%), organooxygen compounds (7.65%), and prenol lipids (6.59%), followed by benzene and substituted derivatives (5.09%), steroids and steroid derivatives (4.79%), indoles and derivatives (2.69%), and flavonoids (1.95%) (Figure 3A). Notably, lipid-related classes (fatty acyls, glycerophospholipids, and prenol lipids) collectively represented a substantial fraction of the altered metabolome, underscoring the prominence of peripheral lipid dysregulation in CFTO-LOAD. Z-score analysis revealed significant alterations in plasma fatty acid and derivative concentrations in AD patients relative to CHC (Figure 3B). Specifically, dodecanoic acid was significantly elevated, whereas arachidonic acid was markedly reduced in AD plasma (Figure 3B), reflecting a shift toward saturated fatty acid accumulation and polyunsaturated fatty acid depletion. To explore potential co-regulation among these lipid alterations, we performed Pearson’s correlation analysis among the significantly changed fatty acids and derivatives. The resulting correlation matrix heatmap (Figure 3C) visualizes pairwise correlations, with red and blue colors denoting positive and negative associations, respectively, and circle size reflecting the absolute correlation coefficient magnitude. Notably, arachidonic acid correlated positively with sphinganine 1-phosphate (*r* = 0.51, *P*_*adj*_ < 0.001) and negatively with dodecanoic acid (*r* = −0.57, *P*_*adj*_ < 0.001; Figure 3C), suggesting coordinated remodeling of the sphingolipid–polyunsaturated fatty acid axis and an inverse relationship between saturated and polyunsaturated fatty acid pools in AD peripheral metabolism. Parallel Z-score analysis of amino acids and their derivatives revealed similarly marked alterations in AD plasma (Figure 3D). L-leucine was significantly elevated, whereas L-arginine was significantly decreased (*P*_*adj*_ < 0.05; Figure 3D), indicating dysregulated branched-chain amino acid metabolism and impaired nitric oxide biosynthesis capacity in CFTO-LOAD. Pearson’s correlation analysis among the altered amino acids and derivatives revealed extensive inter-metabolite co-regulation (Figure 3E). Of particular note, L-leucine exhibited a strong positive correlation with 4-(3-methylbut-2-enyl)-L-tryptophan (*r* = 0.76, *P*_*adj*_ < 0.001), whereas L-arginine correlated negatively with L-alanine (*r* = −0.53, *P*_*adj*_ < 0.001; Figure 3E). These patterns suggest coordinated perturbations in branched-chain amino acid–tryptophan cross-talk and reciprocal changes in the urea cycle–alanine shuttle, which may have implications for neurotransmitter synthesis and systemic nitrogen homeostasis in AD.

**FIGURE 4:**
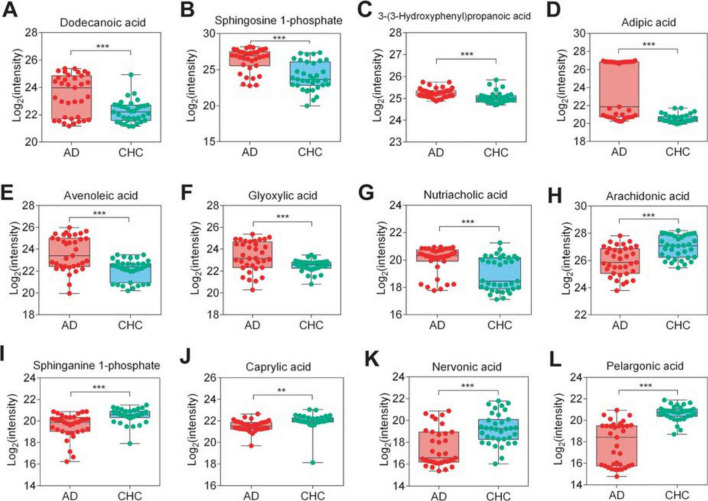
Significant differences in the plasma levels of twelve representative fatty acids and its derivatives between Alzheimer’s disease (AD) and cognitively healthy control (CHC) samples. **(A–L)** Normalized intensity of 12 representative fatty acids and their derivatives, including dodecanoic acid, sphingosine 1-phosphate, 3-(3-hydroxyphenyl) propanoic acid, adipic acid, avenoleic acid, glyoxylic acid, nutriacholic acid, arachidonic acid, sphinganine 1-phosphate, caprylic acid, nervonic acid, and pelargonic acid, in the plasma of 35 AD patients and 35 CHC samples. The abundances of the twelve DAMs were Log_2_-transformed. Samples between the two groups were compared using independent *t*-tests. *P*-values were FDR-corrected using BH method. **P_*adj*_ < 0.01, ***P_*adj*_ < 0.001.

Box plots show that the levels of dodecanoic acid (VIP = 1.04, Log_2_FC = 1.58, *P*_*adj*_ < 0.001), sphingosine 1-phosphate (VIP = 1.29, Log_2_FC = 1.71, *P*_*adj*_ < 0.001), 3-(3-hydroxyphenyl)propanoic acid (VIP = 1.33, Log_2_FC = 0.22, *P*_*adj*_ < 0.001), adipic acid (VIP = 1.38, Log_2_FC = 5.21, *P*_*adj*_ < 0.001), avenoleic acid (VIP = 1.39, Log_2_FC = 1.91, *P*_*adj*_ < 0.001), glyoxylic acid (VIP = 1.08, Log_2_FC = 1.28, *P*_*adj*_ < 0.01), and nutriacholic acid (VIP = 1.27, Log_2_FC = 0.81, *P*_*adj*_ < 0.001) in the plasma of AD patients were significantly increased compared with the CHC group (Figures 4A–G), while the levels of arachidonic acid (VIP = 1.40, Log_2_FC = −0.91, *P*_*adj*_ < 0.001), sphinganine 1-phosphate (VIP = 1.33, Log_2_FC = −0.73, *P*_*adj*_ < 0.001), caprylic acid (VIP = 1.08, Log_2_FC = −0.50, *P*_*adj*_ < 0.01), nervonic acid (VIP = 1.17, Log_2_FC = −1.31, *P*_*adj*_ < 0.001), and pelargonic acid (VIP = 2.00, Log_2_FC = −1.92, *P*_*adj*_ < 0.001) were significantly decreased in AD group (Figures 4H–L). In addition, we also observed other fatty acids and their derivatives, including acetylenedicarboxylic acid (VIP = 1.50, Log_2_FC = 0.51, *P*_*adj*_ < 0.001), 2-aminoisobutyric acid (VIP = 1.05, Log_2_FC = 3.77, *P*_*adj*_ < 0.001), 4-hydroxycyclohexylcarboxylic acid (VIP = 1.01, Log_2_FC = 2.20, *P*_*adj*_ < 0.001), 5-decenoic acid (VIP = 1.32, Log_2_FC = 0.68, *P*_*adj*_ < 0.001), 8-amino-7-oxononanoic acid (VIP = 1.45, Log_2_FC = 0.58, *P*_*adj*_ < 0.001), 8-methylthio-2-octanoic acid (VIP = 1.02, Log_2_FC = 0.25, *P*_*adj*_ < 0.01), 8(R)-hydroperoxylinoleic acid (VIP = 1.26, Log_2_FC = 2.23, *P*_*adj*_ < 0.001), fukiic acid (VIP = 1.85, Log_2_FC = 3.97, *P*_*adj*_ < 0.001), oxoglutaric acid (VIP = 1.58, Log_2_FC = 2.90, *P*_*adj*_ < 0.001), hexadecanedioic acid (VIP = 1.16, Log_2_FC = −0.57, *P*_*adj*_ < 0.001), 2-hydroxyhexadecanoic acid (VIP = 1.71, Log_2_FC = −0.86, *P*_*adj*_ < 0.001), 5Z-dodecenoic acid (VIP = 1.18, Log_2_FC = −0.52, *P*_*adj*_ < 0.001), (2’E, 4’Z, 8E)-colneleic acid (VIP = 1.75, Log_2_FC = −0.60, *P*_*adj*_ < 0.001), (9E)-10-nitrooctadecenoic acid (VIP = 1.25, Log_2_FC = −0.85, *P*_*adj*_ < 0.001), traumatic acid (VIP = 1.95, Log_2_FC = −2.11, *P*_*adj*_ < 0.001), and L-xylonic acid (VIP = 1.44, Log_2_FC = −0.44, *P*_*adj*_ < 0.001), whose concentrations in the plasma of AD patients were significantly altered (Supplementary Figures 2A–P).

**FIGURE 5:**
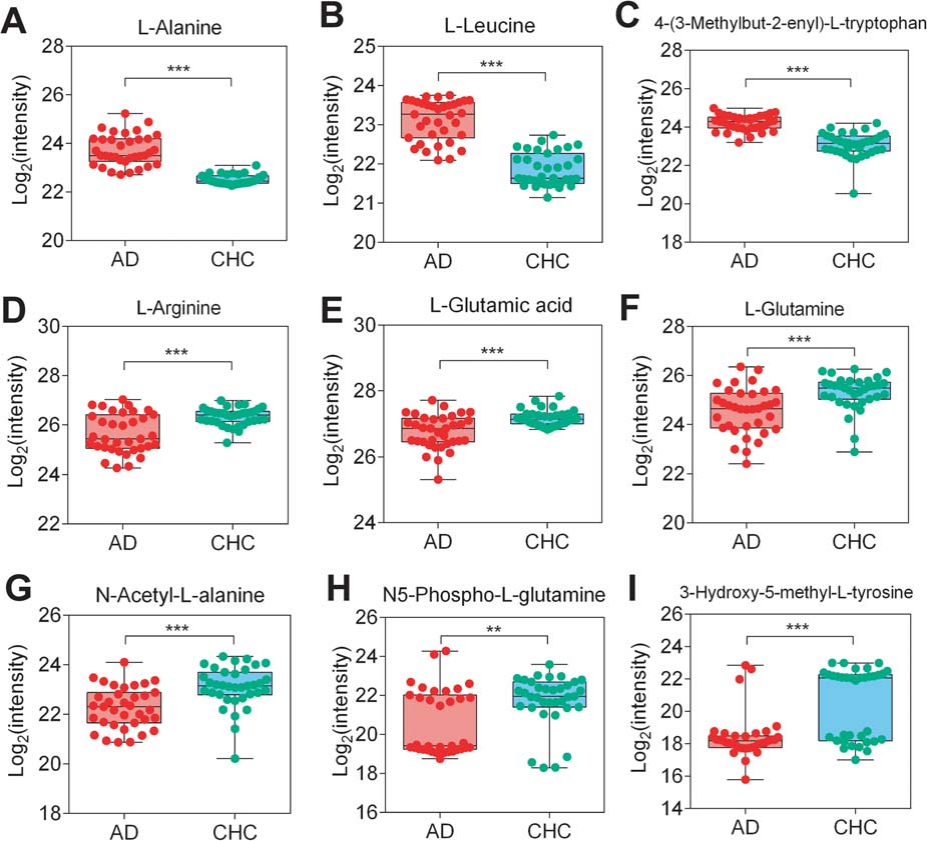
Relative concentrations of nine representative amino acids and its derivatives in the plasma of Alzheimer’s disease (AD) were significantly differed from that of CHC group. **(A–I)** Normalized intensity of nine representative amino acids and its derivatives, including L-alanine, L-leucine, 4-(3-methylbut-2-enyl)-L-tryptophan, L-arginine, L-glutamic acid, L-glutamine, N-acetyl-L-alanine, N5-phospho-L-glutamine, and 3-hydroxy-5-methyl-L-tyrosine, in the plasma of 35 AD patients and 35 CHC samples. The abundances of the nine DAMs were Log_2_-transformed. Samples between the two groups were compared using independent *t*-tests. *P*-values were FDR-corrected using BH method. **P_*adj*_ < 0.01, ***P_*adj*_ < 0.001.

In addition, we also observed significant changes in the levels of amino acids and their derivatives in the plasma of AD patients. For example, compared with the CHC group, the concentrations of L-alanine (VIP = 1.89, Log_2_FC = 1.34, *P*_*adj*_ < 0.001), L-leucine (VIP = 1.88, Log_2_FC = 2.42, *P*_*adj*_ < 0.001), and 4-(3-methylbut-2-enyl)-L-tryptophan (VIP = 1.71, Log_2_FC = 2.10, *P*_*adj*_ < 0.001) were significantly increased in the plasma of AD patients (Figures 5A–C), while the concentrations of L-arginine (VIP = 1.10, Log_2_FC = −0.51, *P*_*adj*_ < 0.001), L-glutamic acid (VIP = 1.33, Log_2_FC = −0.35, *P*_*adj*_ < 0.001), L-glutamine (VIP = 1.17, Log_2_FC = −0.60, *P*_*adj*_ < 0.001), N-acetyl-L-alanine (VIP = 1.05, Log_2_FC = −0.79, *P*_*adj*_ < 0.001), N5-phospho-L-glutamine (VIP = 1.02, Log_2_FC = −0.55, *P*_*adj*_ < 0.01), and 3-hydroxy-5-methyl-L-tyrosine (VIP = 1.16, Log_2_FC = −2.06, *P*_*adj*_ < 0.001) were significantly decreased in AD group (Figures 5D–I).

**FIGURE 6:**
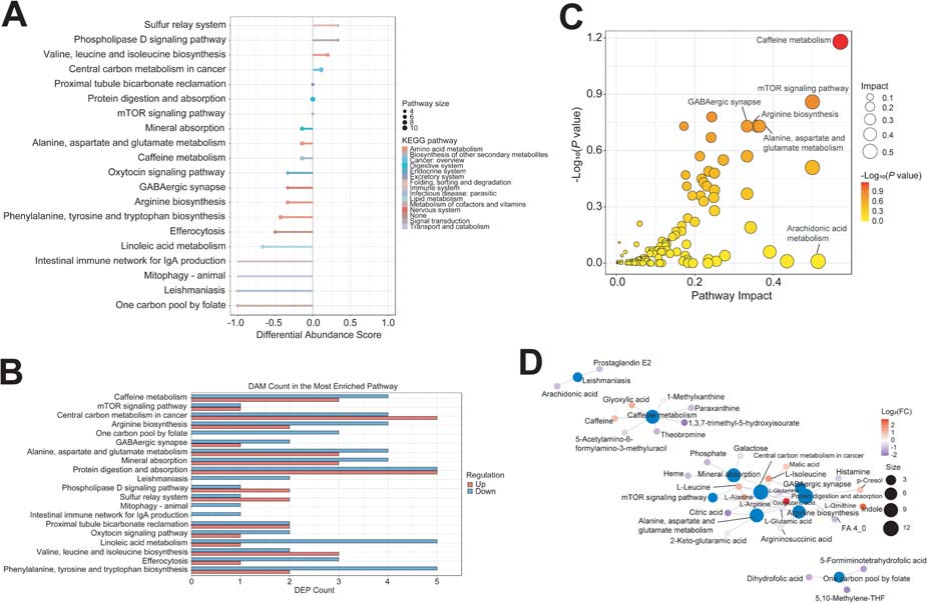
KEGG pathway enrichment analysis of 875 DAMs identified in AD samples. **(A)** Differential abundance score plot demonstrates top20 ranked pathways enriched by the 875 DAMs in AD patients, such as GABAergic synapse, arginine biosynthesis, and linoleic acid metabolism. **(B)** Bar graph shows multiple pathways enriched by up-regulated and down-regulated DAMs, respectively. **(C)** Bubble chart presents most top-ranking pathways, such as GABAergic synapse, mTOR signaling pathway, arginine biosynthesis, alanine, aspartate and glutamate metabolism, enriched by 875 DAMs in the peripheral system of AD patients. **(D)** Network diagram exhibits representative pathways enriched by certain metabolites, for example, L-glutamine and L-glutamic acid was participated in GABAergic synapse pathway in AD patients.

The KEGG pathway enrichment analysis results showed that these DAMs were mainly enriched in lipid metabolism, amino acid metabolism, cofactors and vitamins metabolism, nervous system, digestive system, endocrine system, excretory system, immune system, signal transduction, transport and catabolism, folding, sorting and degradation pathways (Figure 6A). In particular, the pathways of lipid and amino acid metabolism include linoleic acid metabolism, valine, leucine, and isoleucine biosynthesis, alanine, aspartate and glutamate metabolism, phenylalanine, tyrosine and tryptophan biosynthesis, and arginine biosynthesis; the pathways of the nervous and immune systems include GABAergic synapse and intestinal immune network for IgA production; the pathways of signal transduction include phospholipase D signaling pathway, mTOR signaling pathway, and oxytocin signaling pathway (Figures 6A, B). Among the above enriched signaling pathways, the most significant were caffeine metabolism, mTOR signaling pathway, GABAergic synapse, arginine, aspartate and glutamate metabolism, and so on (Figure 6C). Among them, L-glutamine and L-glutamic acid were involved in multiple signaling pathways including GABAergic synapse, arginine, aspartate and glutamate metabolism, and central carbon metabolism (Figure 6D).

### 3.3 Immune inflammatory responses and Aβ and Tau pathological changes in the peripheral system of CFTO-LOAD patients

**FIGURE 7:**
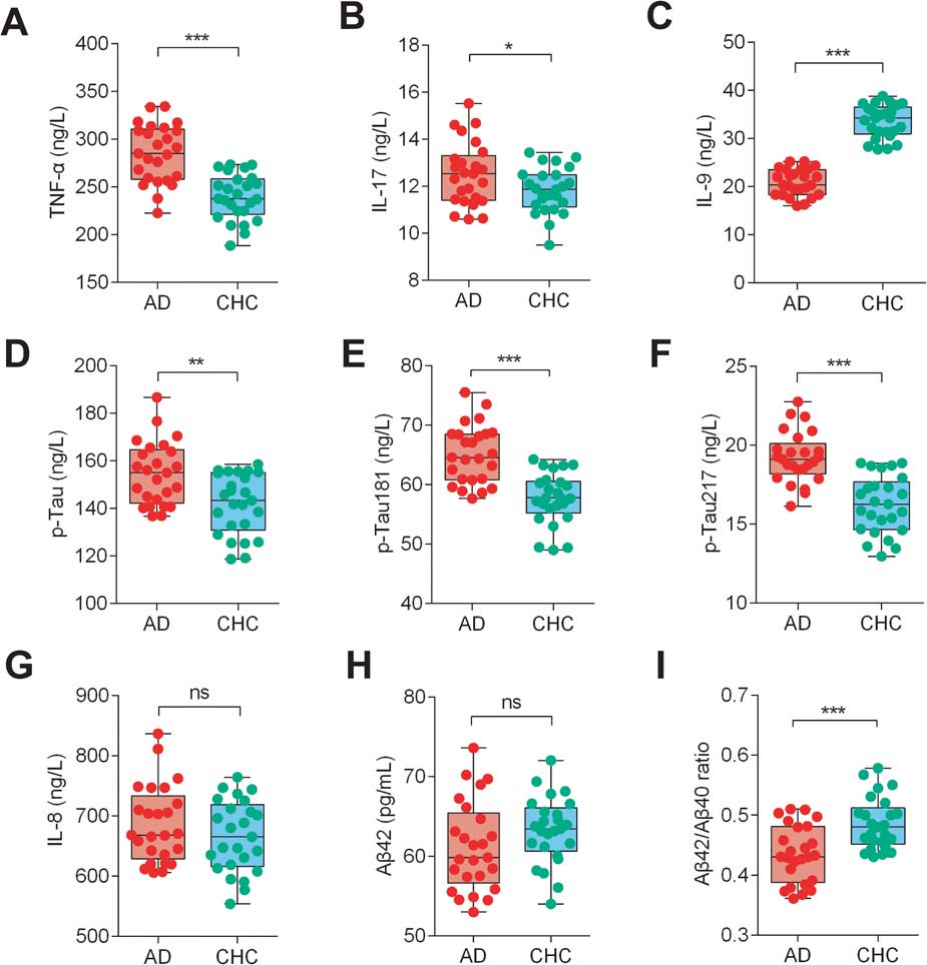
Plasma levels of nine representative cytokines, chemokines, soluble Tau and amyloid β-protein (Aβ) in Alzheimer’s disease (AD) patients were significantly changed relative to cognitively healthy control (CHC). **(A–I)** Circulating levels of TNF-α, IL-17, IL-9, p-Tau, p-Tau181, p-Tau217, IL-8, Aβ42, and Aβ42/Aβ40 ratio in the plasma of AD patients were significantly altered compared to that of CHC. Samples between the two groups were compared using independent *t*-tests. *P*-values were FDR-corrected using BH method. *P_*adj*_ < 0.05, **P_*adj*_ < 0.01, ***P_*adj*_ < 0.001.

To elucidate the native immune inflammatory response changes and the level changes of soluble Tau and Aβ in the peripheral system of CFTO-LOAD patients, the concentrations of immune inflammatory markers (e.g., cytokines TNF-α, IL-17, and IL-9, and chemokine IL-8), Tau pathological markers (including p-Tau, p-Tau181, and p-Tau217), and Aβ pathological markers (including Aβ40, Aβ42, and Aβ42/Aβ40 ratio) in the plasma of CFTO-LOAD and CHC samples we detected by ELISA, and corrected for age to exclude the influence of age on inflammatory factor and pathology marker levels. Box plots show that the concentrations of pro-inflammatory cytokines TNF-α and IL-17 were significantly increased in the plasma of CFTO-LOAD patients compared with the CHC group (*P*_*adj*_ < 0.05; Figures 7A, B), while the level of IL-9 was significantly decreased in AD (*P*_*adj*_ < 0.001; Figure 7C). Compared with the CHC group, the concentrations of soluble total phosphorylated Tau protein (i.e., p-Tau) and site-specific phosphorylated Tau proteins, such as p-Tau181 and p-Tau217, were significantly increased in the plasma of CFTO-LOAD patients (*P*_*adj*_ < 0.01; Figures 7D–F). Although we observed no significant changes in the concentrations of chemokine IL-8 and soluble Aβ42 in the plasma of CFTO-LOAD patients relative to CHC (Figures 7G, H), while the Aβ42/Aβ40 ratio was significantly reduced in CFTO-LOAD patients (*P_*adj*_* < 0.001; Figure 7I). These results indicated that the peripheral immune inflammatory response in CFTO-LOAD patients was significantly enhanced, with significant pathological features such as phosphorylated Tau and the Aβ42/Aβ40 ratio.

### 3.4 Significant correlations between peripheral amino acid metabolism, immuno-inflammatory responses, and soluble Tau and Aβ in CFTO-LOAD patients

Peripheral amino acid metabolism and systemic inflammation are bidirectionally linked (Yang et al., 2023). Proinflammatory cytokines such as TNF-α and IL-17 modulate amino acid uptake and catabolism; conversely, amino acids including tryptophan, arginine, and glutamine serve as critical regulators of immune cell activation and cytokine production (McGaha et al., 2012; Murray, 2016; Yang et al., 2023). To investigate these immuno-metabolic interactions and their relationship with AD-related pathology, we performed linear regression analyses between differential amino acids, proinflammatory cytokines, and soluble Tau and Aβ species.

**FIGURE 8:**
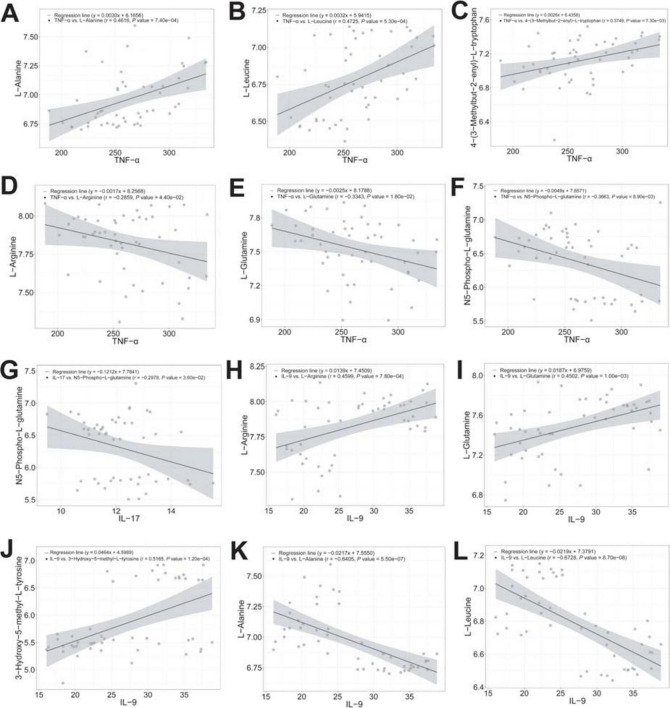
Changes of seven amino acids and its derivatives levels were significantly correlated to altered concentrations of TNF-α, IL-17, and IL-9. **(A–F)** Significant correlation between the level changes of TNF-α and altered abundances of L-alanine **(A)**, L-leucine **(B)**, 4-(3-methylbut-2-enyl)-L-tryptophan **(C)**, L-arginine **(D)**, L-glutamine **(E)**, and N5-phosphol-L-glutamine **(F)** in AD patients. **(G)** Significant correlation between the concentration changes of IL-17 and altered levels of N5-phospho-L-glutamine. **(H–L)** Significant correlation between the level changes of IL-9 and altered contents of L-arginine **(H)**, L-glutamine **(I)**, 3-hydroxy-5-methyl-L-tyrosine **(J)**, L-alanine **(K)**, and L-leucine **(L)**. Statistical importance was determined by the Pearson’s correlation (r) and probability (*P*). Gray area around the straight line indicates 95% confidence interval.

Correlation analysis revealed robust associations between amino acid disturbances and proinflammatory cytokines (Figure 8). Elevated plasma TNF-α was positively correlated with increased L-alanine (*r* = 0.46, *P_*adj*_* < 0.001), L-leucine (*r* = 0.47, *P_*adj*_* < 0.001), and 4-(3-methylbut-2-enyl)-L-tryptophan (*r* = 0.37, *P_*adj*_* < 0.01; Figures 8A–C), and negatively correlated with decreased L-arginine (*r* = −0.29, *P_*adj*_* < 0.05), L-glutamine (*r* = −0.33, *P_*adj*_* < 0.05), and N5-phospho-L-glutamine (*r* = −0.37, *P_*adj*_* < 0.01; Figures 8D–F). These patterns align with the known capacity of TNF-α to induce indoleamine 2,3-dioxygenase (IDO)-mediated tryptophan degradation and to modulate glutamine and arginine utilization in activated immune cells (Munn and Mellor, 2013; Zhang et al., 2024), thereby establishing a mechanistic link between peripheral amino acid pools and TNF-α-driven inflammatory tone. IL-17 also showed a significant negative correlated with reduced N5-phospho-L-glutamine (*r* = −0.30, *P_*adj*_* < 0.05; Figure 8G), suggesting that Th17-mediated signaling may alter glutamine bioavailability. In addition, decreased IL-9 was positively correlated with reduced L-arginine (*r* = 0.46, *P_*adj*_* < 0.001), L-glutamine (*r* = 0.45, *P_*adj*_* < 0.01), and 3-hydroxy-5-methyl-L-tyrosine (*r* = 0.52, *P_*adj*_* < 0.001; Figures 8H–J), and negatively correlated with elevated L-alanine (*r* = −0.64, *P_*adj*_* < 0.001) and L-leucine (*r* = −0.67, *P_*adj*_* < 0.001; Figures 8K, L).

**FIGURE 9:**
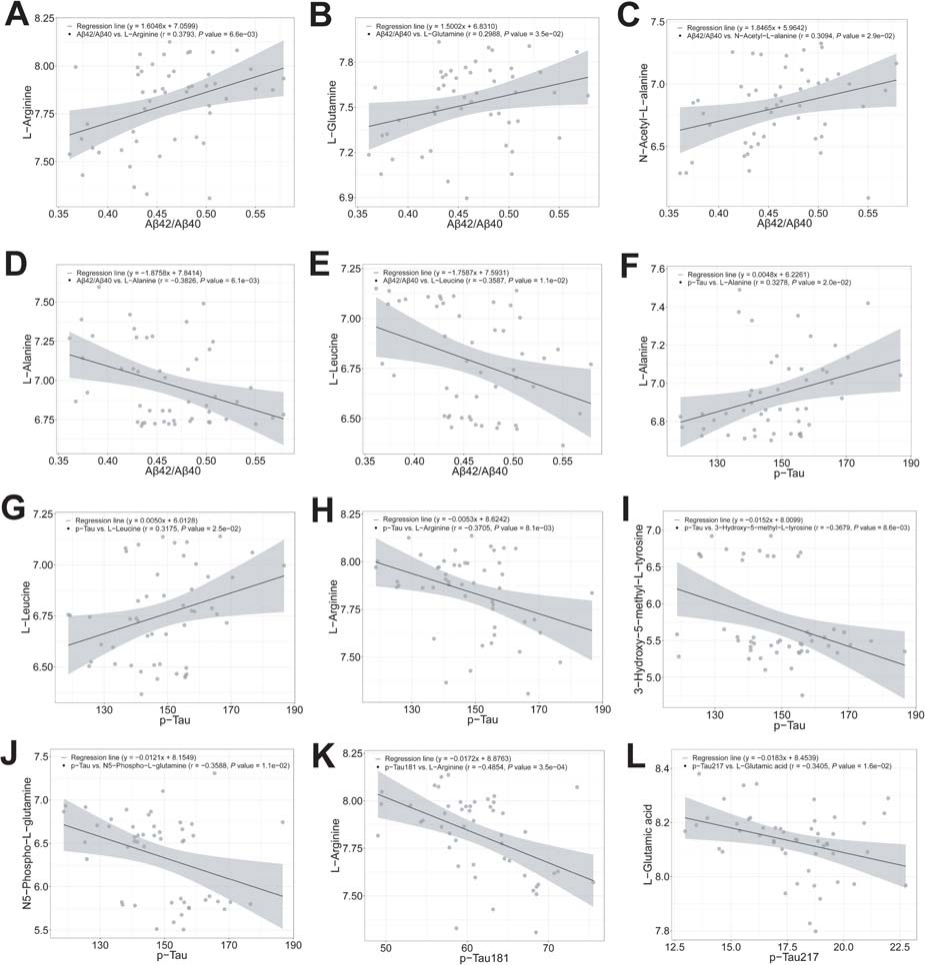
Alterations of eight amino acids and its derivatives levels were significantly correlated to changed Aβ42/Aβ40 ratio, and concentrations of phosphorylated Tau. **(A–E)** Significant correlation between the changes of Aβ42/Aβ40 ratio and altered concentrations of L-arginine **(A)**, L-glutamine **(B)**, N-acetyl-L-alanine **(C)**, L-alanine **(D)**, L-leucine **(E)**. **(F–J)** Significant correlation between the level changes of p-Tau and altered abundances of L-alanine **(F)**, L-leucine **(G)**, L-arginine **(H)**, 3-hydroxy-5-methyl-L-tyrosine **(I)**, N5-phospho-L-glutamine **(J)**. **(K)** Significant correlation between the level changes of p-Tau181 and altered concentrations of L-arginine. **(L)** Significant correlation between the level changes of p-Tau217 and altered abundances of L-glutamic acid. Statistical importance was determined by the Pearson’s correlation (r) and probability (*P*). Gray area around the straight line indicates 95% confidence interval.

We next examined the relationship between amino acid alterations and AD-related pathological markers. A decreased Aβ42/Aβ40 ratio was positively correlated with reduced L-arginine (*r* = 0.38, *P_*adj*_* < 0.01), L-glutamine (*r* = 0.30, *P_*adj*_* < 0.05), and N-acetyl-L-alanine (*r* = 0.31, *P_*adj*_* < 0.05; Figures 9A–C), and negatively correlated with elevated L-alanine (*r* = −0.38, *P_*adj*_* < 0.01) and L-leucine (*r* = −0.36, *P_*adj*_* < 0.05; Figures 9D, E). Conversely, elevated soluble p-Tau was positively correlated with increased L-alanine (*r* = 0.33, *P_*adj*_* < 0.05) and L-leucine (*r* = 0.32, *P_*adj*_* < 0.05; Figures 9F, G), and negatively correlated with decreased L-arginine (*r* = −0.37, *P_*adj*_* < 0.01), 3-hydroxy-5-methyl-L-tyrosine (*r* = −0.37, *P_*adj*_* < 0.01), and N5-Phospho-L-glutamine (*r* = −0.36, *P_*adj*_* < 0.05; Figures 9H–J). Elevated soluble p-Tau181 was negatively correlated with reduced L-arginine (*r* = −0.49, *P_*adj*_* < 0.001), while elevated soluble p-Tau217 was negatively correlated with reduced L-glutamic acid (*r* = −0.34, *P_*adj*_* < 0.05; Figures 9K, L). Additional analyses revealed that p-Tau181 was positively correlated with L-alanine (*r* = 0.54, *P_*adj*_* < 0.001), L-leucine (*r* = 0.50, *P_*adj*_* < 0.001), and 4-(3-methylbut-2-enyl)-L-tryptophan (*r* = 0.40, *P_*adj*_* < 0.01; Supplementary Figures 3A–C), and negatively correlated with L-glutamine (*r* = −0.32, *P_*adj*_* < 0.05) and L-glutamic acid (*r* = −0.32, *P_*adj*_* < 0.05; Supplementary Figures 3D, E). Similarly, p-Tau217 correlated positively with L-alanine (*r* = 0.52, *P_*adj*_* < 0.001), L-leucine (*r* = 0.51, *P_*adj*_* < 0.001), and 4-(3-methylbut-2-enyl)-L-tryptophan (*r* = 0.51, *P_*adj*_* < 0.001; Supplementary Figures 3F–H), and negatively with L-glutamine (*r* = −0.41, *P_*adj*_* < 0.01; Supplementary Figures 3I). Collectively, these findings indicate that peripheral amino acid dysregulation in CFTO-LOAD is tightly coupled to TNF-α- and IL-17-mediated inflammatory responses and to Tau and Aβ pathological burden, supporting an immuno-metabolic axis that may drive disease progression.

### 3.5 Significant correlations between peripheral fatty acid metabolism, immuno-inflammatory responses, and soluble Tau and Aβ in CFTO-LOAD patients

Fatty acids and their derivatives serve not only as energy substrates but also as critical signaling molecules and precursors for pro- and anti-inflammatory lipid mediators (Dyall et al., 2022). In the context of AD, peripheral fatty acid imbalances may both drive and respond to systemic inflammation through the generation of eicosanoids, resolvins, and related oxylipins (de Wit et al., 2021; Shinto et al., 2022). To elucidate the interplay between lipid dysregulation and inflammatory status in our cohort, we examined correlations between the significantly altered fatty acids and proinflammatory cytokines.

**FIGURE 10:**
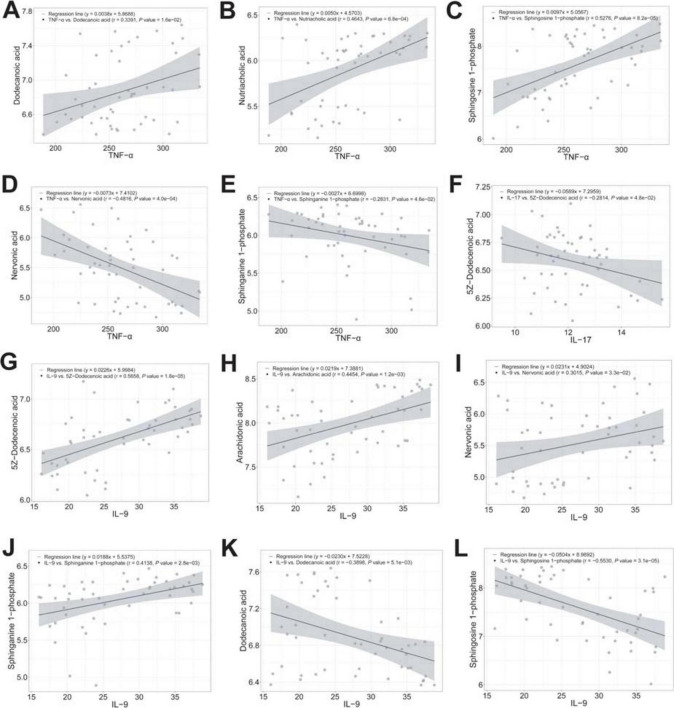
Changes of seven fatty acids and its derivatives concentrations were significantly correlated to altered levels of TNF-α, IL-17, and IL-9. **(A–E)** Significant correlation between the level changes of TNF-α and altered abundances of dodecanoic acid **(A)**, nutriacholic acid **(B)**, sphingosine 1-phosphate **(C)**, nervonic acid **(D)**, and sphinganine 1-phosphate **(E)** in AD patients. **(F)** Significant correlation between the level changes of IL-17 and altered contents of 5Z-dodecenoic acid. **(G–L)** Significant correlation between the level changes of IL-9 and altered concentrations of 5Z-dodecenoic acid **(G)**, arachidonic acid **(H)**, nervonic acid **(I)**, sphinganine 1-phosphate **(J)**, dodecanoic acid **(K)**, and sphingosine 1-phosphate **(L)**. Statistical importance was determined by the Pearson’s correlation (r) and probability (*P*). Gray area around the straight line indicates 95% confidence interval.

We identified robust correlations between fatty acid alterations and cytokine levels (Figure 10). Elevated TNF-α was positively correlated with increased dodecanoic acid (*r* = 0.34, *P_*adj*_* < 0.05), nutriacholic acid (*r* = 0.46, *P_*adj*_* < 0.001), and sphingosine 1-phosphate (*r* = 0.53, *P_*adj*_* < 0.001; Figures 10A–C), and negatively correlated with decreased nervonic acid (*r* = −0.48, *P_*adj*_* < 0.001) and sphinganine 1-phosphate *(r* = −0.28, *P_*adj*_* < 0.05; Figures 10D, E). These associations suggest that TNF-α signaling may promote saturated fatty acid and sphingolipid mediator accumulation while depleting neuroprotective very-long-chain fatty acids in the periphery. IL-17 was negatively correlated with reduced 5Z-dodecenoic acid (*r* = −0.28, *P_*adj*_* < 0.05; Figure 10F), indicating that Th17-driven inflammation may be associated with altered monounsaturated fatty acid status. Decreased IL-9 was positively correlated with reduced 5Z-dodecenoic acid (*r* = 0.57, *P_*adj*_* < 0.001), arachidonic acid (*r* = 0.45, *P_*adj*_* < 0.01), nervonic acid (*r* = 0.30, *P_*adj*_* < 0.05), and sphinganine 1-phosphate (*r* = 0.41, *P_*adj*_* < 0.01; Figures 10G–J), and negatively correlated with elevated dodecanoic acid (*r* = −0.39, *P_*adj*_* < 0.01) and sphingosine 1-phosphate (*r* = −0.55, *P_*adj*_* < 0.001; Figures 10K, L).

**FIGURE 11:**
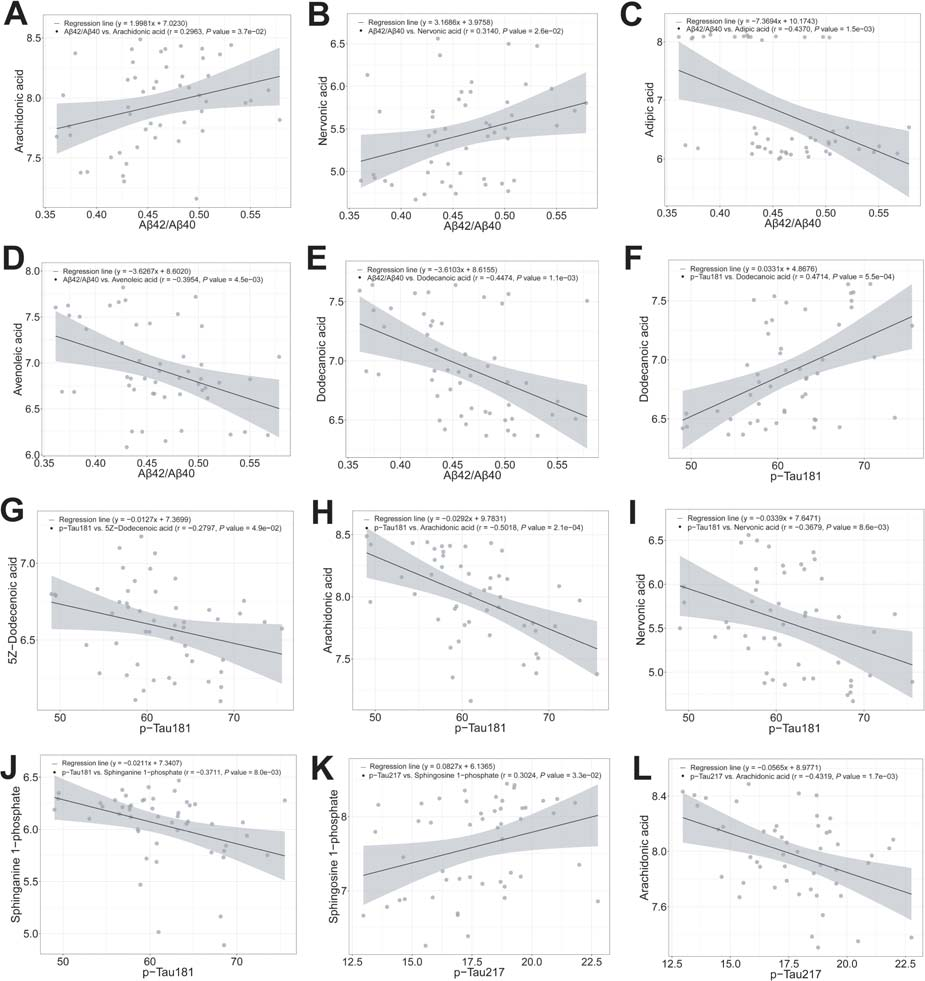
Changes of eight fatty acids and its derivatives levels were significantly correlated to changed Aβ42/Aβ40 ratio, and concentrations of phosphorylated Tau. **(A–E)** Significant correlation between the reduction of Aβ42/Aβ40 ratio and altered concentrations of arachidonic acid **(A)**, nervonic acid **(B)**, adipic acid **(C)**, avenoleic acid **(D)**, and dodecanoic acid **(E)**. **(F–J)** Significant correlation between the increase of p-Tau181 level and changed abundances of dodecanoic acid **(F)**, 5Z-dodecenoic acid **(G)**, arachidonic acid **(H)**, nervonic acid **(I)**, sphinganine 1-phosphate **(J)**. **(K,L)** Significant correlation between the up-regulation of p-Tau217 level and changed concentrations of sphingosine 1-phosphate **(K)** and arachidonic acid **(L)**. Statistical importance was determined by the Pearson’s correlation (r) and probability (*P*). Gray area around the straight line indicates 95% confidence interval.

We next assessed the relationship between fatty acid disturbances and AD-related pathological markers. A decreased Aβ42/Aβ40 ratio was positively correlated with reduced arachidonic acid (*r* = 0.30, *P_*adj*_* < 0.05) and nervonic acid (*r* = 0.31, *P_*adj*_* < 0.05; Figures 11A, B), and negatively correlated with elevated adipic acid (*r* = −0.44, *P_*adj*_* < 0.01), avenoleic acid (*r* = −0.40, *P_*adj*_* < 0.01), and dodecanoic acid (*r* = −0.45, *P_*adj*_* < 0.01; Figures 11C–E). Elevated soluble p-Tau181 was positively correlated with increased dodecanoic acid (*r* = 0.47, *P_*adj*_* < 0.001; Figure 11F), and negatively correlated with decreased 5Z-dodecenoic acid (*r* = −0.28, *P_*adj*_* < 0.05), arachidonic acid (*r* = −0.50, *P_*adj*_* < 0.001), nervonic acid (*r* = −0.37, *P_*adj*_* < 0.01), and sphinganine 1-phosphate (*r* = −0.37, *P_*adj*_* < 0.01; Figures 11G–J). Elevated soluble p-Tau217 was positively correlated with increased sphingosine 1-phosphate (*r* = 0.30, *P_*adj*_* < 0.05; Figure 11K), and negatively correlated with decreased arachidonic acid (*r* = −0.43, *P_*adj*_* < 0.01; Figure 11L). Additional analyses revealed that a decreased Aβ42/Aβ40 ratio was positively correlated with reduced 2-hydroxyhexadecanoic acid (*r* = 0.35, *P_*adj*_* < 0.05) and (9E)-10-nitrooctadecenoic acid (*r* = 0.36, *P_*adj*_* < 0.05; Supplementary Figures 4A, B), and negatively correlated with elevated 2-aminoisobutyric acid (*r* = −0.37, *P_*adj*_* < 0.01), 4-hydroxycyclohexylcarboxylic acid (*r* = −0.36, *P_*adj*_* < 0.01), 8-amino-7-oxononanoic acid (*r* = −0.28, *P_*adj*_* < 0.05), and 8(R)-hydroperoxylinoleic acid (*r* = −0.32, *P_*adj*_* < 0.05; Supplementary Figures 4C–F). Elevated soluble p-Tau was positively correlated with increased sphingosine 1-phosphate (*r* = 0.38, *P_*adj*_* < 0.01; Supplementary Figure 4G). Elevated soluble p-Tau181 was positively correlated with increased adipic acid (*r* = 0.47, *P_*adj*_* < 0.001) and avenoleic acid (*r* = 0.42, *P_*adj*_* < 0.01; Supplementary Figures 4H, I). Collectively, these findings demonstrate that peripheral fatty acid dysregulation in CFTO-LOAD is intimately coupled to TNF-α- and IL-17-mediated proinflammatory signaling and to Tau and Aβ pathological burden, supporting a lipid-immuno-inflammatory axis in disease pathophysiology.

## 4 Discussion

In recent years, metabolic reprogramming and immuno-metabolic regulation have emerged as critical determinants of AD pathogenesis (Asimakidou et al., 2025; Baik et al., 2019; Bettcher et al., 2021; Haage and De Jager, 2022; Kamer, 2010; Lepiarz-Raba et al., 2023; Peralta Ramos et al., 2025; Shippy and Ulland, 2020; Testa et al., 2016; Yang et al., 2021). Pharmacological interventions (e.g., anti-Aβ monoclonal antibodies (Mangialasche et al., 2010)) and neuromodulatory therapies [e.g., repetitive transcranial magnetic stimulation (Chou et al., 2022; Koch et al., 2018; Moussavi et al., 2024) and transcranial direct current stimulation (Chen et al., 2022; Hou et al., 2024; Meléndez et al., 2023)] can substantially alter patients’ metabolic profiles and immune-inflammatory status. Consequently, studies involving treated cohorts may obscure the native metabolic signatures associated with spontaneous disease progression. By focusing exclusively on treatment-naïve, late-stage AD outpatients, the present study captures the unperturbed peripheral metabolic landscape and its interplay with systemic inflammation and neurodegenerative pathology.

Our untargeted metabolomic profiling revealed pronounced peripheral metabolic reprogramming in CFTO-LOAD patients, characterized by coordinated alterations in lipid, fatty acid, and amino acid pools. Specifically, we observed accumulation of saturated medium-chain fatty acids (e.g., dodecanoic acid) and certain sphingolipid derivatives (e.g., sphingosine 1-phosphate), alongside depletion of polyunsaturated fatty acids (e.g., arachidonic acid) and very-long-chain species (e.g., nervonic acid). Concurrently, amino acid profiling indicated disrupted nitrogen metabolism, with marked changes in branched-chain amino acids (BCAAs), aromatic amino acids, and glutamine/glutamate balance. These patterns align with previous AD metabolomic investigations (Basun et al., 1990; Ma et al., 2025); however, the exclusive focus on treatment-naïve, late-stage, first-time outpatients provides a distinctive window into the metabolic derangements that accompany advanced spontaneous disease progression, unconfounded by therapeutic intervention.

Beyond cataloging metabolic alterations, our findings elucidate their functional integration with systemic immune-inflammatory responses. Elevated plasma TNF-α and IL-17, together with reduced IL-9, indicate a skewed proinflammatory milieu in CFTO-LOAD. The observed correlations between amino acid disturbances and cytokine profiles suggest the existence of a regulated immuno-metabolic axis. Rather than passive epiphenomena, these metabolic shifts may actively shape immune cell function and inflammatory tone. For instance, diminished L-arginine bioavailability may compromise nitric oxide (NO) synthesis, thereby impairing macrophage efferocytosis and reducing Aβ clearance capacity (Bronte and Zanovello, 2005; Canè et al., 2025; Ma et al., 2021; Rath et al., 2014; Rodriguez et al., 2017; Virarkar et al., 2013; Vural et al., 2009). This interpretation is reinforced by pathway enrichment analysis identifying significant enrichment in efferocytosis and mitophagy, suggesting compromised immune cell clearance functions in late-stage AD. Furthermore, L-arginine is indispensable for T-cell activation and differentiation (Bronte and Zanovello, 2005; Bronte et al., 2003; Canè et al., 2025; Rodriguez et al., 2007; Werner et al., 2017); its depletion may suppress CD4^+^ and CD8^+^ T-cell responses, contributing to systemic immune dysregulation.

Concurrently, elevated L-leucine and L-alanine may drive immune cell metabolic reprogramming via mTOR signaling activation. mTOR orchestrates macrophage polarization toward the proinflammatory M1 phenotype and promotes CD4^+^ T-cell differentiation into IL-17-secreting Th17 cells (Byles et al., 2013; Chi, 2012; Covarrubias et al., 2015; Kim et al., 2013; Korn et al., 2007; Schnell et al., 2023; Weichhart et al., 2015; Zeng and Chi, 2017), thereby amplifying the inflammatory cascade. The observed enrichment of the mTOR signaling pathway among AD-related DAMs supports its activation in CFTO-LOAD. The reduction in L-glutamine and L-glutamic acid further supports this paradigm, as glutamine serves as a primary energy substrate for immune cells and critically regulates macrophage polarization (Hu et al., 2024; Jiang et al., 2022). Diminished glutamine availability may precipitate bioenergetic failure in immune cells, exacerbating neuroinflammation and compromising Aβ and Tau clearance (Pinky Wali et al., 2025; Yin et al., 2016; Yuan et al., 2024). Taken together, these findings suggest that peripheral amino acid imbalances in CFTO-LOAD are active participants in shaping the proinflammatory immune microenvironment.

Fatty acid alterations also exhibited intimate coupling with inflammatory signaling. Arachidonic acid, a precursor for prostaglandins and leukotrienes, plays a central role in propagating inflammatory responses (Jang et al., 2020; Kuehl and Egan, 1980). Its depletion in CFTO-LOAD may reflect enhanced consumption via the cyclooxygenase and lipoxygenase pathways, potentially driven by chronic TNF-α stimulation. Conversely, elevated dodecanoic acid (lauric acid) may potentiate Toll-like receptor signaling and inflammasome activation in macrophages (Gaete et al., 2023; Rodríguez et al., 2025), further amplifying cytokine release. The reduction in nervonic acid, a critical very-long-chain monounsaturated fatty acid for myelin integrity, may compromise neuronal membrane fluidity and receptor signaling, indirectly affecting microglial surveillance and neuroinflammatory tone (Phung et al., 2024). These lipid-mediated mechanisms illustrate how peripheral fatty acid dysregulation can simultaneously influence systemic immunity and central nervous system homeostasis.

The interplay between metabolic disturbances and Aβ/Tau pathology extends beyond immune modulation. Amino acid imbalances may directly affect neuronal bioenergetics and protein homeostasis. Reduced L-arginine and L-glutamine could impair mitochondrial oxidative phosphorylation and ATP generation, creating a cellular environment conducive to Tau hyperphosphorylation and aggregation. Elevated BCAAs may alter brain insulin signaling and amyloidogenic processing, potentially shifting the balance toward Aβ production over clearance. Fatty acid derivatives, particularly sphingosine-1-phosphate and its precursors, function as potent signaling lipids that regulate neuroinflammation, blood-brain barrier permeability, and glial activation. Thus, the peripheral metabolic profile in CFTO-LOAD may reflect—and potentially drive—a self-amplifying cycle wherein metabolic stress fuels neuroinflammation, which in turn exacerbates metabolic dysfunction and proteinopathy.

From a translational perspective, the identified metabolic signatures may offer candidate biomarkers for LOAD staging and progression monitoring. However, we approach this proposition with due caution. Peripheral metabolite alterations are frequently observed across multiple metabolic and neurodegenerative conditions, including type 2 diabetes mellitus, obesity, cardiovascular disease, and other dementias (e.g., vascular dementia and Lewy body dementia). The metabolic changes reported here may therefore reflect shared pathophysiological processes—such as chronic low-grade inflammation, insulin resistance, and mitochondrial dysfunction—rather than AD-specific pathology. Moreover, the diagnostic utility of these analytes would likely be restricted to specific demographic and pathophysiological subgroups, given the heterogeneity of AD and the profound influence of comorbidities, dietary patterns, and genetic background on peripheral metabolism. Future studies must rigorously evaluate the discriminative capacity of these metabolites against appropriate disease controls, assess their performance across diverse ethnic and age-stratified populations, and establish longitudinal trajectories to determine whether they predict conversion from mild cognitive impairment to LOAD or track disease progression. Until such validation is achieved, these metabolites should be regarded as exploratory candidates requiring extensive cross-disease comparative analysis and prospective cohort verification.

This study has several limitations. First, the modest sample size (35 subjects per group) may limit statistical power and increase the risk of false-positive discoveries. Despite FDR correction, the identification of 875 DAMs from a relatively small sample carries a substantial risk of overfitting and Type I error inflation, given the high dimensionality of untargeted metabolomics data relative to statistical power. Consequently, some metabolite features may lack reproducibility across independent cohorts. Validation in larger, multicenter cohorts is essential, and targeted validation of prioritized biomarkers (e.g., L-arginine, L-leucine, arachidonic acid, dodecanoic acid, and sphingosine 1-phosphate) in an independent cohort is strongly recommended and has been incorporated into our future research plan. Second, our analysis was restricted to peripheral plasma; it does not capture the metabolic milieu of active lesion areas in postmortem brain tissue or cerebrospinal fluid. Readers must exercise caution when interpreting these plasma biomarkers: while these differential metabolites reflect peripheral systemic metabolic alterations, they cannot directly or comprehensively represent CNS metabolism or neuroinflammation. The blood-brain barrier, cerebral metabolic autonomy, distinct microglial versus peripheral macrophage populations, and compartment-specific immune responses create a substantial biological boundary between peripheral and central inflammatory states. Future investigations should integrate central and peripheral metabolomics to delineate whether these plasma alterations mirror or merely correlate with CNS pathology. Furthermore, although all blood samples were collected under standardized fasting conditions, we did not systematically record or control for habitual dietary patterns, caloric intake, or specific nutrient consumption in the preceding weeks to months. Dietary habits profoundly influence peripheral lipid and amino acid profiles; therefore, the metabolic signatures observed may partially reflect dietary heterogeneity rather than disease-specific alterations. Future studies will incorporate detailed dietary questionnaires and standardized nutritional assessments to exclude dietary confounders. Third, the cross-sectional design strictly precludes causal inference. All observed associations between metabolites, cytokines, and AD pathology markers are correlational, and readers should refrain from inferring causality or therapeutic utility from these data alone. Longitudinal studies tracking metabolite fluctuations across preclinical, prodromal, and dementia stages are warranted to establish their value as prognostic indicators. Fourth, although we identified moderate correlations between metabolites, cytokines, and soluble Tau/Aβ markers, causality remains unproven. Functional studies utilizing *in vitro* immune cell models, AD transgenic animals, and multi-omics integration (proteomics, transcriptomics) are required to dissect the molecular mechanisms by which specific metabolites regulate macrophage, T-cell, and glial functions, and to determine whether targeted metabolic interventions (e.g., L-arginine or arachidonic acid modulation) can ameliorate pathology. Fifth, readers must interpret the reported plasma biomarker correlations with caution. The majority of Pearson correlation coefficients fall within the moderate range (*r* = 0.30–0.50), indicating that these metabolite-cytokine and metabolite-pathology associations explain only 9%–25% of shared variance. While statistically significant after multiple-testing correction, the biological and clinical significance of these moderate correlations remains uncertain. These associations may represent indirect, confounded, or epiphenomenal relationships rather than direct mechanistic links. Future investigations utilizing cellular co-culture models and AD transgenic animal experiments are warranted to elucidate whether these biomarkers engage in genuine biological interactions and to assess their potential clinical relevance beyond statistical association. Finally, as emphasized above, the biomarker potential of these analytes requires cautious interpretation pending comprehensive specificity testing against metabolic and neurodegenerative disease controls.

## 5 Conclusion

This study delineated the peripheral amino acid and fatty acid metabolic profiles in treatment-naïve, late-stage Alzheimer’s disease patients at first clinical presentation, and uncovered their complex interplay with immune-inflammatory responses, Aβ, and Tau pathology. These findings provide a theoretical basis and novel insights for identifying candidate prognostic biomarkers and developing therapeutic strategies for late-stage AD. By characterizing the native metabolic signatures of treatment-naïve late-stage patients, we offer a new conceptual perspective and a valuable reference for understanding late-stage AD pathophysiology. Collectively, these results inform the development of novel biomarkers and support therapeutic strategies targeting immuno-metabolic regulation.

## Data Availability

The original contributions presented in this study are included in the article/Supplementary material, further inquiries can be directed to the corresponding authors.

## Footnotes

1
http://www.hmdb.ca

2
http://www.mzcloud.org

3
http://massbank.jp

4
http://www.lipidmaps.org

5
https://mona.fiehnlab.ucdavis.edu

6
https://metlin.scripps.edu

7
www.metaboanalyst.ca
