# Supplementary material for: Peripheral metabolomic profiling reveals lipid and amino acid alterations associated with immuno-inflammatory responses in treatment-naïve late-onset Alzheimer’s disease
Source: Front Aging Neurosci. 2026 Jun 23;18:1858299. doi: 10.3389/fnagi.2026.1858299 (PMC13337820; doi:10.3389/fnagi.2026.1858299)
Supplement: Supplementary file 1 [file Data_Sheet_1.docx]

**Supplementary figures**


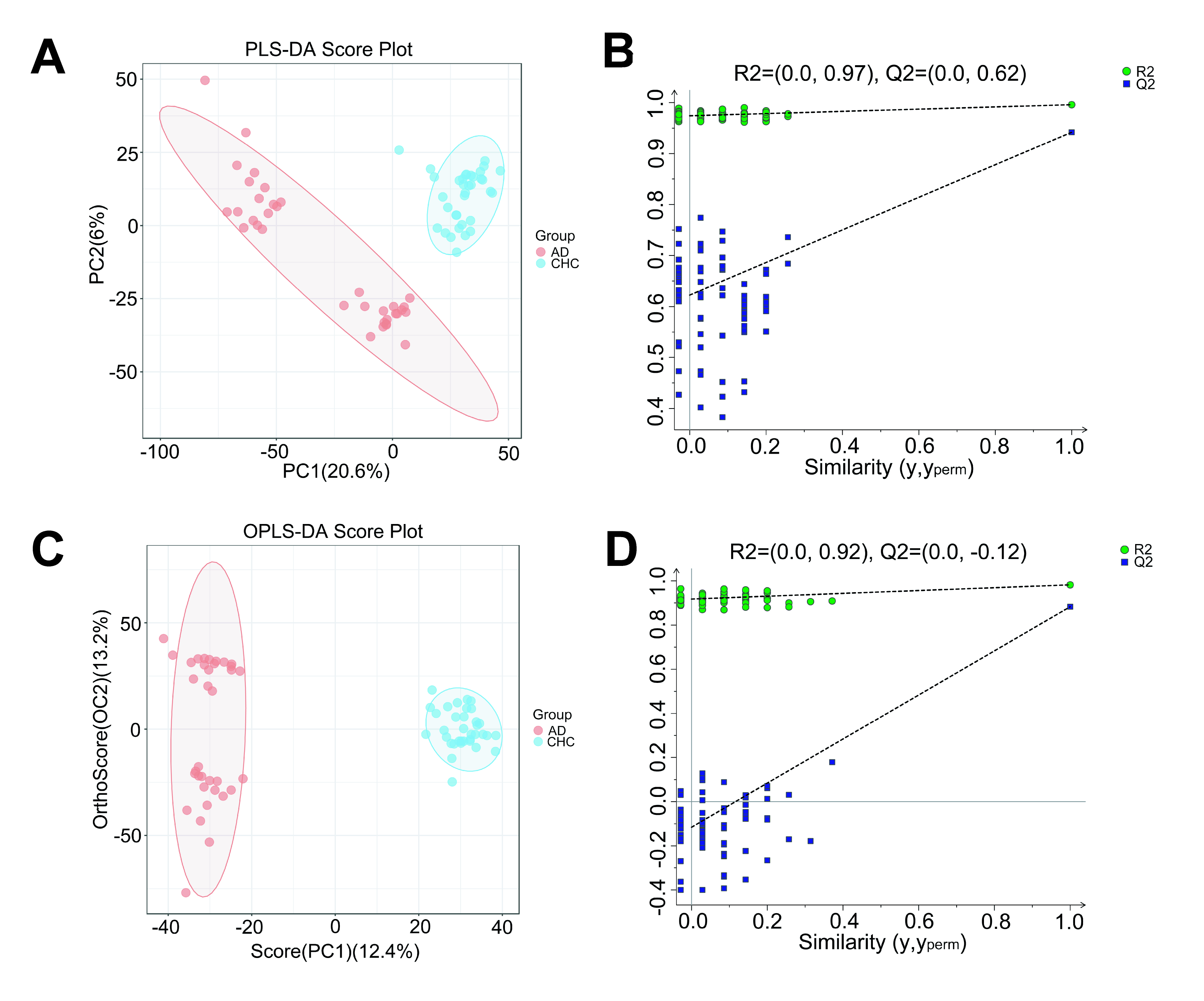


**Figure S1.** **Supervised multivariate statistical analysis in anionic mode demonstrate clear separation between AD and CHC samples.** (**A**) and (**B**) PLS-DA analysis results of 35 AD and 35 CHC samples in anionic mode. The model interpretability R2X = 0.318, and R2Y = 0.996, model predictability Q2 = 0.942. (**C**) and (**D**) OPLS-DA analysis results of 35 AD and 35 CHC samples in anionic mode. The model interpretability R2X = 0.297, and R2Y = 0.982, model predictability Q2 = 0.883.


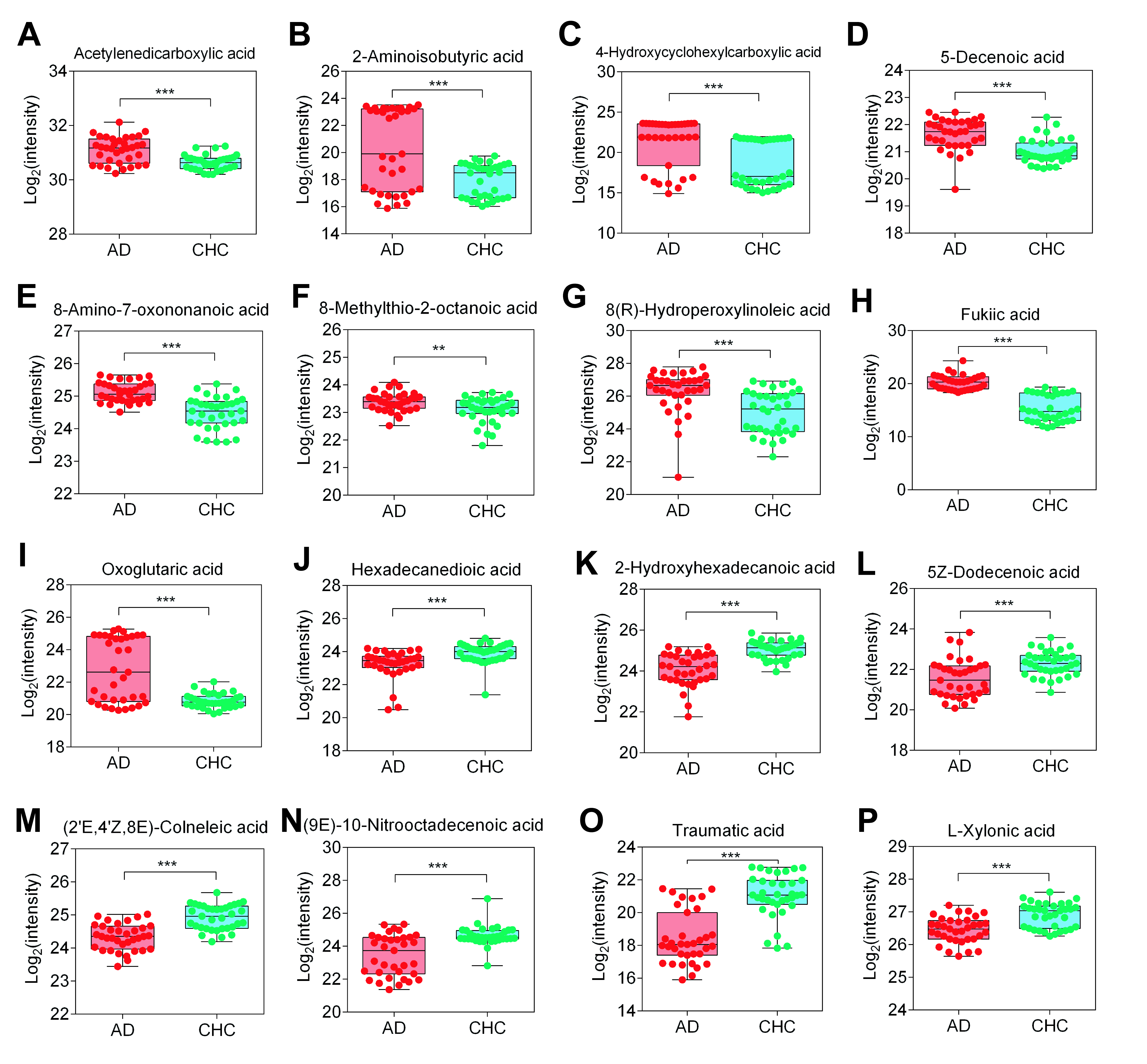


**Figure S2. Significant differences in the plasma levels of sixteen representative fatty acids and its derivatives between AD and CHC samples.** (**A**-**P**) Normalized intensity of sixteen representative fatty acids and their derivatives, including acetylenedicarboxylic acid, 2-aminoisobutyric acid, 4-hydroxycyclohexylcarboxylic acid, 5-decenoic acid, 8-amino-7-oxononanoic acid, 8-methylthio-2-octanoic acid, 8(R)-hydroperoxylinoleic acid, fukiic acid, oxoglutaric acid, hexadecanedioic acid, 2-hydroxyhexadecanoic acid, 5Z-dodecenoic acid, (2’E,4’Z,8E)-colneleic acid, (9E)-10-nitrooctadecenoic acid, traumatic acid, and L-xylonic acid, in the plasma of 35 AD patients and 35 CHC samples. The abundances of the sixteen DAMs were Log_2_-transformed. Samples between the two groups were compared using independent t-tests. *P* values were FDR-corrected using BH method. **: *P_adj_* < 0.01, ***: *P_adj_* < 0.001.


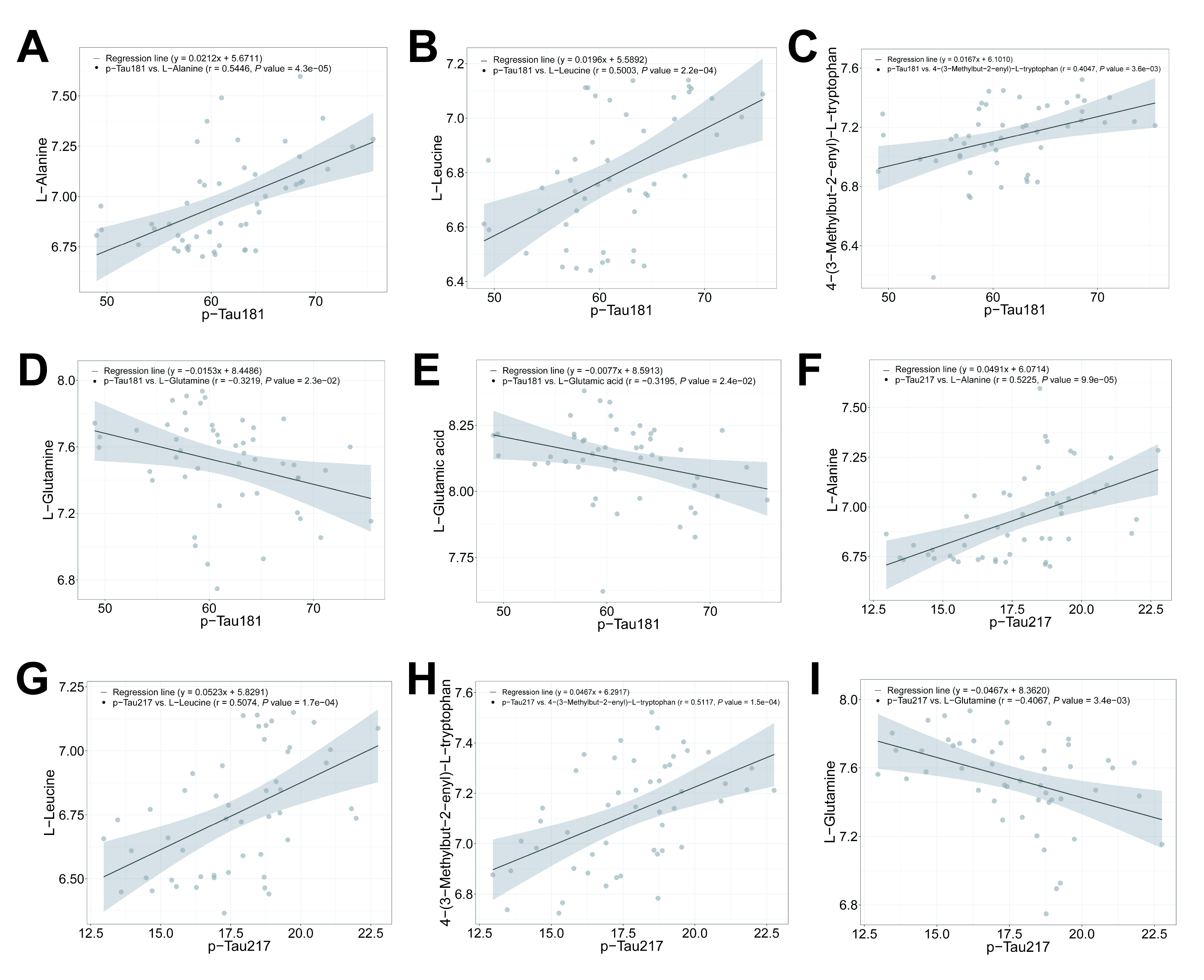


**Figure S3. Changes of five amino acids and its derivatives levels were significantly correlated to altered concentrations of phosphorylated Tau.** (**A**-**E**) Significant correlation between the changes of p-Tau181 levels and altered abundances of L-alanine (**A**), L-leucine (**B**), 4-(3-methylnut-2-enyl)-L-tryptophan (**C**), L-glutamine (**D)**, and L-glutamic acid (**E**) in AD patients. (**F**-**I**) Significant correlation between the elevation of p-Tau217 levels and changed concentrations of L-alanine (**F**), L-leucine (**G**), 4-(3-methylnut-2-enyl)-L-tryptophan (**H**), and L-glutamine (**I)**. Statistical importance was determined by the Pearson’s correlation (r) and probability (*P*). Gray area around the straight line indicates 95% confidence interval.


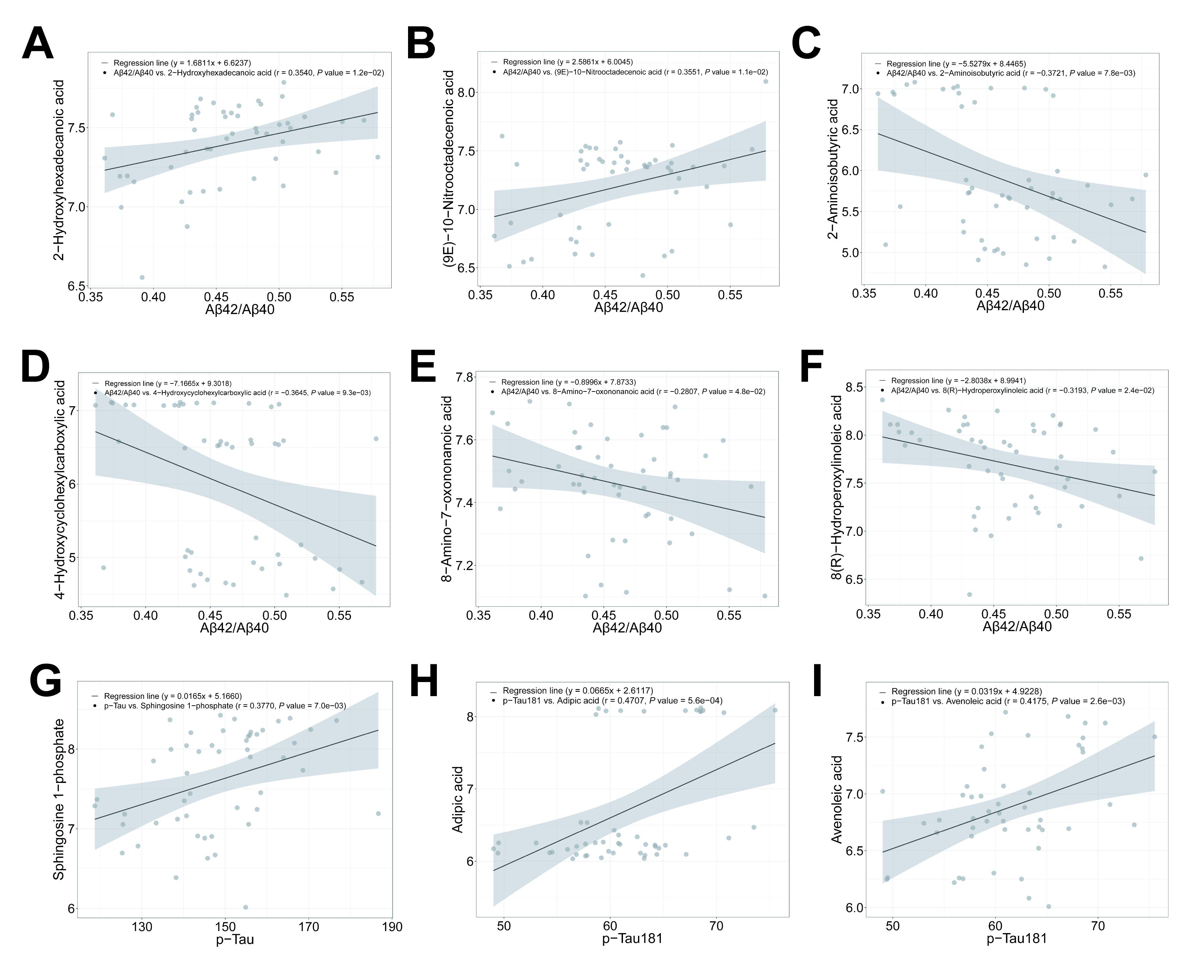


**Figure S4. Changes of nine fatty acids and its derivatives levels were significantly correlated to changed Aβ42/Aβ40 ratio, and concentrations of phosphorylated Tau.** (**A**-**F**) Significant correlation between the reduction of Aβ42/Aβ40 ratio and changed concentrations of 2-hydroxyhexadecanoic acid (**A**), (9E)-10-nitrooctadecenoic acid (**B**), 2-aminoisobutyric acid (**C**), 4-hydroxycyclohexylcarboxylic acid (**D**), 8-amino-7-oxononanoic acid (**E**), and 8(R)-hydroperoxylinoleic acid (**F**). (**G**) Significant correlation between the increase of p-Tau level and up-regulated level of sphingosine 1-phosphate. (**H**) and (**I**) Significant correlation between the up-regulation of p-Tau181 level and changed concentrations of adipic acid (**H**) and avenoleic acid (**I**). Statistical importance was determined by the Pearson’s correlation (r) and probability (*P*). Gray area around the straight line indicates 95% confidence interval.
