# Supplementary material for: Peripheral metabolomic profiling reveals lipid and amino acid alterations associated with immuno-inflammatory responses in treatment-naïve late-onset Alzheimer’s disease
Source: Front Aging Neurosci. 2026 Jun 23;18:1858299. doi: 10.3389/fnagi.2026.1858299 (PMC13337820; doi:10.3389/fnagi.2026.1858299)
Supplement: Supplementary file 2 [file Data_Sheet_2.docx]

**Supplementary table 1**

**Table S1** The characteristics of CFTO-LOAD and CHC recruited in this study

| **Characteristics** | **CFTO-LOAD** | **CHC** | ***P* value** |
| --- | --- | --- | --- |
| Number | 35 | 35 | NA |
| Gender, male/female | 10/25 | 11/24 | NA |
| Age (years), mean ± SD. | 80.40 ± 8.32 | 73.11 ± 4.01 | < 0.001 |
| MMSE, mean ± SD. | 6.43 ± 7.54 | 28.46 ± 1.20 | < 0.001 |
| AD8, mean ± SD. | 7.66 ± 0.64 | 1.29 ± 1.13 | < 0.001 |
| EOAD/Familial AD dementia | 0 | 0 | NA |
| Other neurodegenerative or mental disorders (such as Parkinson’s disease, depression and schizophrenia) | 0 | 0 | NA |
| Diagnosed cancers/tumors | 0 | 0 | NA |
| Hypertension | 0 | 0 | NA |
| Diabetes mellitus | 0 | 0 | NA |
| Hyperlipidemia | 0 | 0 | NA |
| Other autoimmune or inflammatory illnesses | 0 | 0 | NA |
| Active bacterial, fungal, or viral infections | 0 | 0 | NA |
| Obesity (BMI ≥ 30.0) | 0 | 0 | NA |
| Smoking | 0 | 0 | NA |
| Drinking | 0 | 0 | NA |
| Treatment of immune-modulating drugs within 6 months | 0 | 0 | NA |

CFTO-LOAD, Chinese first-time outpatients with late-onset Alzheimer’s disease; CHC, cognitively healthy control; MMSE: Mini-Mental State Examination; AD8: Alzheimer’s Disease-8; EOAD: Early-onset Alzheimer’s disease; BMI: body mass index; SD, standard deviation; NA, not available.
